# Prognosis Relevance of Serum Cytokines in Pancreatic Cancer

**DOI:** 10.1155/2015/518284

**Published:** 2015-08-04

**Authors:** Carolina Torres, Ana Linares, Maria José Alejandre, Rogelio J. Palomino-Morales, Octavio Caba, Jose Prados, Antonia Aránega, Juan R. Delgado, Antonio Irigoyen, Joaquina Martínez-Galán, Francisco M. Ortuño, Ignacio Rojas, Sonia Perales

**Affiliations:** ^1^Department of Biochemistry and Molecular Biology I, University of Granada, 18071 Granada, Spain; ^2^Department of Health Sciences, University of Jaen, 23071 Jaen, Spain; ^3^Department of Human Anatomy and Embryology, University of Granada, 18012 Granada, Spain; ^4^Oncology Service, Virgen de las Nieves Hospital, 18014 Granada, Spain; ^5^Department of Computer Architecture and Computer Technology (CITIC-UGR), University of Granada, 18071 Granada, Spain

## Abstract

The overall survival of patients with pancreatic ductal adenocarcinoma is extremely low. Although gemcitabine is the standard used chemotherapy for this disease, clinical outcomes do not reflect significant improvements, not even when combined with adjuvant treatments. There is an urgent need for prognosis markers to be found. The aim of this study was to analyze the potential value of serum cytokines to find a profile that can predict the clinical outcome in patients with pancreatic cancer and to establish a practical prognosis index that significantly predicts patients' outcomes. We have conducted an extensive analysis of serum prognosis biomarkers using an antibody array comprising 507 human cytokines. Overall survival was estimated using the Kaplan-Meier method. Univariate and multivariate Cox's proportional hazard models were used to analyze prognosis factors. To determine the extent that survival could be predicted based on this index, we used the leave-one-out cross-validation model. The multivariate model showed a better performance and it could represent a novel panel of serum cytokines that correlates to poor prognosis in pancreatic cancer. B7-1/CD80, EG-VEGF/PK1, IL-29, NRG1-beta1/HRG1-beta1, and PD-ECGF expressions portend a poor prognosis for patients with pancreatic cancer and these cytokines could represent novel therapeutic targets for this disease.

## 1. Introduction

Pancreatic ductal adenocarcinoma (PDAC) accounts for only 2.68% of all cancers, but it represents the fourth leading cancer-related death worldwide just remaining after lung and bronchus, prostate, and colorectum cancers in men and after lung and bronchus, breast, and colorectum cancers in women [1]. The dreadful prognosis of patients with this disease, less than 5% reaching 5 years of survival after diagnosis, is due to the little impact of the available chemotherapy on the course of the disease and to tumor metastasis at presentation. The development of the disease is a result of a complex and does not yet fully understood process encompassing the accumulation of mutations and the alteration of multiple pathways. This could partly explain the clinical heterogeneity of this disease and the great difference seen in the outcomes between individual patients. Thereby, there is a trend towards tailored therapies to specific genetic characteristics of individual tumors, not only for PDAC but also for the majority of the cancers [2, 3]. Throughout past years there has not been remarkable survival improvement in PDAC patients; consequently it is urgent that novel biomarkers are identified for PDAC in order to reduce its mortality rate [4, 5].

As defined by the NIH Biomarker Working Group, a biological marker (biomarker) is a characteristic that is objectively measured and evaluated as an indicator of normal biological processes, pathogenic processes, or pharmacologic responses to a therapeutic intervention [6]. In PDAC, three types of biomarkers are desirable: those that help in the detection of the disease onset (*diagnosis biomarkers*); those that predict responses to treatments (*predictive biomarkers*); and those that forecast the likely course of the disease, including survival and recurrence pattern in the absence of therapy (*prognosis biomarkers*). Finding a biomarker or a panel of biomarkers that would be able to predict the clinical impact of a chemotherapy regimen even before it is initiated is highly warranted to (1) identify those patients more likely to benefit from aggressive therapies; (2) reduce risks of useless side effects and help to set expectations for doctors and patients; and (3) make attempt to apply new combination of therapies or individualized treatment protocols according to their expected responses. In addition, markers that display prognosis significance also offer the potential to become emergent therapeutic targets and novel strategies in the management of PDAC [7, 8]. In the recent years, an extensive research has been focused on the discovery of prognosis biomarkers for PDAC using immunohistochemistry, Western Blot, PCR, miRNA, proteomics, or DNA methylation based methods amongst others [9–16].

Here we have focused on those inflammatory mediators that may constitute useful prognosis biomarkers for PDAC detection. Altered levels of circulating inflammatory cytokines have been found in cancer patients for nearly every cancer examined, even at early stages of the development, indicating that immune response has an important role during carcinogenesis and that circulating inflammatory markers may be useful cancer biomarkers [17, 18]. Cytokines are signaling molecules that are key mediators of inflammation or immune response. We presume that, due to the extremely important role of microenvironment and desmoplastic reaction in PDAC [19, 20], cytokines expression patterns within the tumor and the surrounding microenvironment could represent potential prognosis biomarkers for PDAC.

The aim of this study was to investigate the prognosis significance of serum cytokines as a reflection of the host response to tumor in PDAC patients. A conditional stepwise algorithm based on likelihood rate analysis according to the Cox's proportional hazard model was used to identify the best combination of significant prognosis factors. An equation was then derived for modeling the survival in our specific cohort. A leave-one-out cross validation was developed to assess the model.

## 2. Material and Methods

### 2.1. Patients and Sample Collection

All patients in the study were diagnosed with PDAC at Hospital Virgen de las Nieves (Granada, Spain) from 2008 to 2011 (*n* = 14). All information from patients, including gender, age, disease grade, and symptoms was recorded. The mean age of the patients was 66 years (range, 41–79 years) with a male to female ratio of 50 : 50. Clinical staging for patients with pancreatic adenocarcinoma was as follows: stage III (28%) and stage IV (72%) (Table 1). PDAC patients had an overall survival time of 12.6 months, all being treated under Gemcitabine + Erlotinib combined therapy following the pattern previously defined by Moore et al. [21]. There was not any history of pancreatitis but 36% had type II diabetes mellitus and 36% were smokers. Blood samples were collected after obtaining the approval of relevant ethics committees and informed consents of donors. Serum samples were collected from 2008 to 2011 using standard procedures at the Oncology Service of Virgen de las Nieves Hospital. Blood samples were obtained from patients diagnosed with PDAC at baseline and at two weeks after initiation of therapy (Gemcitabine + Erlotinib) and also from healthy individuals (14 samples). However, for this study only pretreatment serum samples from patients were considered to propose the prognosis cytokine panel. Serum was obtained after blood centrifugation at 1500 rpm for 10 min at 4°C. Samples were aliquoted and stored at −80°C.

### 2.2. Cytokine Antibody Assay

Soluble proteins in the sera of PDAC patients were measured using a biotin label-based human antibody array (Human Antibody L-series 507 Array (RayBiotech, Norcross, GA, USA)), according to the recommended protocols. Briefly, all samples were biotinylated. Antibodies were immobilized in specific spot locations on glass slides. Incubation of array membranes with biological samples resulted in the binding of cytokines to corresponding antibodies. Signals were visualized using streptavidin-HRP conjugates and colorimetric. Final spot intensities were measured as the original intensities subtracting the background. Data were normalized to the positive controls in the individual slide. The antibody array data is provided in Supplementary Table 3 (see Supplementary Material available online at http://dx.doi.org/10.1155/2015/518284).

### 2.3. Statistical Analysis

All statistics and data analysis were performed using the* IBM SPSS statistic 20 software* or the statistical language R. Quality analysis was performed using the “ArrayQualityMetrics” package in R to eliminate any feasible outlier [22]. Average survival after administration of Gemcitabine and Erlotinib was calculated from the beginning of the treatment to death, in months. For overall survival (OS) analyses, the Kaplan-Meier curve was used as a method that estimates the probability of survival to a given time using proportion of patients who have survived to that time [23]. The OS method has been widely applied in several relevant studies to analyze how well a treatment works [24–26]. The log-rank test was used to determine survival differences between groups. Kaplan-Meier survival curves for individual markers were obtained after dichotomization. The cut-off values for each marker were those which displayed the most significant discrimination between short (<6 months) and long (>6 months) survival. In order to determine the most significant variables contributing to the OS, univariate and multivariate analyses were performed with the Cox's proportional hazard regression model [27] to determine associations between serum cytokines and cancer-related mortality. First, we analyzed associations between death and levels of cytokines in patients before treatment, considering one factor at a time. Second, a multivariate Cox's proportional hazard model was applied. The wrapper analysis was used as the feature selection method, using conditional forward stepwise algorithm based on likelihood rate, as applied in other works [16, 28]. Wrapper methods attempt to jointly select sets of variables with good predictive power for a predictor [29]. Forward selection starts with an empty set and selects the variable that gives the best classification result. Given this first variable, another variable is added that realizes the largest improvement of performance. Variables are added until the performance does not improve [30].

The overall model fit was considered significant based on chi-squared statistic test (*P* < 0.05). Besides, Wald index was shown to determine the weight of each variable in the global model, both uni- and multivariate. The cytokine levels were introduced in the models as continuous parameters and results were expressed as the hazard ratio (HR) or relative risk ratio for one unit change. By analysis of these variables, a prognosis index (PI) that considers the regression coefficients derived by Cox's model of all significant factors was obtained. This model and the PI calculation were carried out according to the equations provided by Cox [27]. Differences were considered significant when *P* < 0.05.

Leave-one-out cross validation (LOOCV) was applied to assess the performance of the prognosis model as the simplest and most widely used method for estimating prediction accuracy [31]. For this validation, one patient was removed from the initial set, leaving a temporary training set and one left-out sample (test sample). The training set was used to obtain the Cox regression model. Subsequently, the PI of the test sample was obtained from the previously performed model. This PI was also applied to classify this patient according to the OS as poor (<6 months) and fair (>6 months) prognosis.

## 3. Results

### 3.1. Survival Analysis of Patients with PDAC

**Figure 1:**
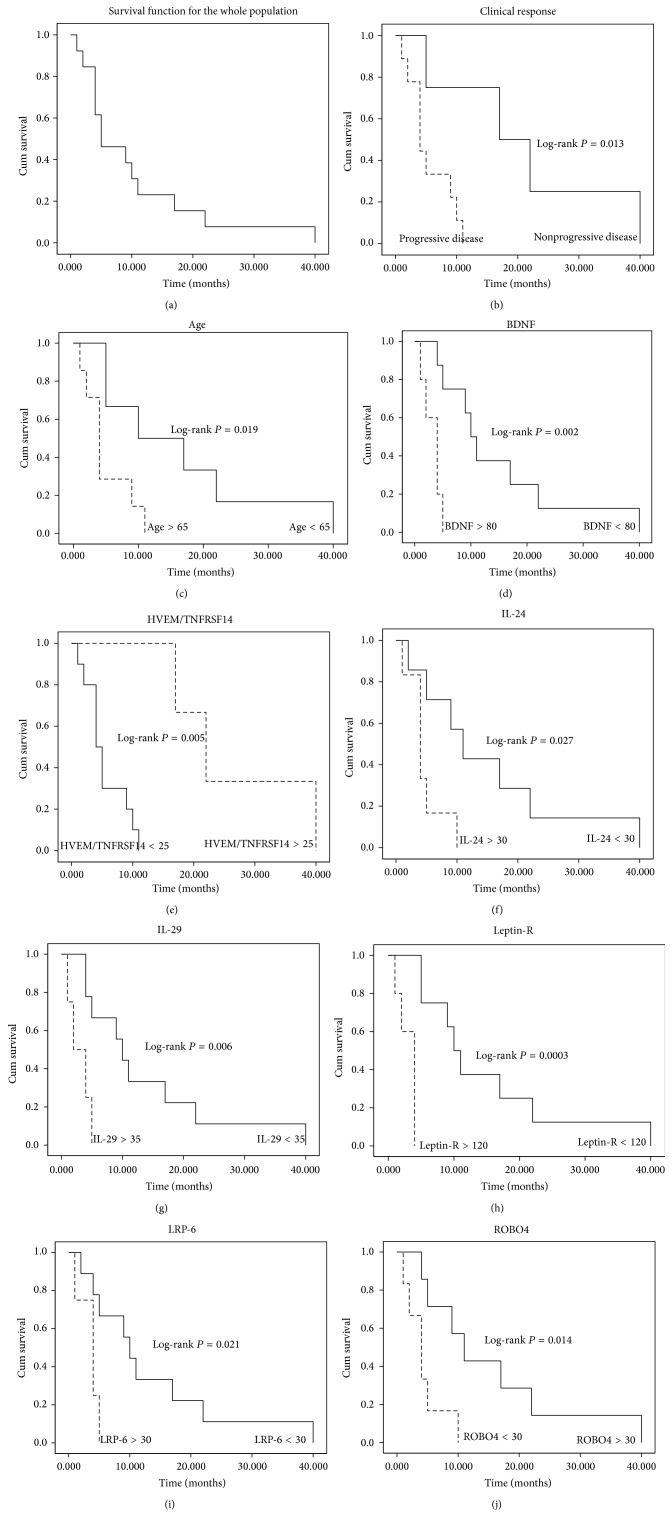
(a) shows Kaplan-Meier disease-specific survival curve for the whole population in the study. The Kaplan-Meier survival curve is defined as the probability of surviving in a given period of time. Each period of time is the interval between two nonsimultaneous terminal events. There were no survival data censored as no information about the survival time of any individual was lost. (b–h) Plots depict Kaplan-Meier survival curves of individual biomarkers tagged as significant prognosis markers: (b) clinical response; (c) age; (d) BDNF; (e) HVEM/TNFRSF14; (f) IL-24; (g) IL-29; (h) leptin-R; (i) LRP-6; and (j) ROBO4. The cut-off values were determined considering those points which maximized the dichotomization between poor and fair prognosis. The *P* values for the log-rank tests are shown for every variable.

Clinical characteristics of the PDAC patients are summarized in Table 1. For the whole study population, the OS rates were 46.15% at 6 months, 23.08% at 12 months, and 7.69% at 24 months. Mean duration of the follow-up for the entire study group was 12 months (range: 1–40 months) and during that time the 100% of the PDAC patients died due to the disease. Survival probabilities were calculated using the Kaplan-Meier method. The survival curve for the whole cohort of patients is shown in Figure 1(a).

### 3.2. Univariate Analysis between Serum Cytokines and Survival

First, a univariate approach was used in this study to identify relevant and independent measurable factors at prognosis that could be associated to a higher risk of PDAC death. Serum levels of cytokines before treatment and clinicopathologic parameters such as age, gender, stage, and clinical response were analyzed. Amongst the clinicopathologic parameters, age and the clinical response (progressive or nonprogressive disease, according to the RECIST criteria [32]) were associated with poor prognosis on univariate analysis (*P* = 0.030 and *P* = 0.013, resp.). Concerning cytokines, at univariate analysis after feature selection, expression levels of BDNF (*P* = 0.034, HR 1.005, 95% CI (1.000–1.009)); HVEM/TNFRSF14 (*P* = 0.039, HR 0.924, 95% (CI 0.858–0.996)); IL-24 (*P* = 0.023, HR 1.041, 95% CI (1.006–1.078)); IL-29 (*P* = 0.021, HR 1.012, 95% CI (1.002–1.023)); leptin R (*P* = 0.018, HR 1.008, 95% CI (1.001–1.015)); LRP-6 (*P* = 0.022 HR 1.027, 95% CI (1.004–1.051)), and ROBO4 (*P* = 0.045 HR 1.002, 95% CI (1.000–1.004)) showed a significant influence on prognosis. Results of the univariate analysis of each cytokine as independent prognosis factors and its beta-coefficients (*β*), hazard ratios (representing the factor by which the hazard changes for each one-unit increase of the cytokine expression), 95% CI (upper and lower limits of the confidence interval with a significance level of 0.05), and *P* values are shown in Table 2. In order to determine survival differences of these individual markers in PDAC patients, Kaplan-Meier survival curves were generated using the cut-off points providing the most significant discrimination in terms of survival between groups (short and long survival). Figures 1(b)–1(j) depict Kaplan-Meier survival plots of individual markers showing significant prognosis differences. For each curve, the log-rank *P* value was provided to show that the differences between the two groups were significant.

### 3.3. Multivariate Analysis between Serum Cytokines and Survival

Despite the fact that often only those statistically significant variables in univariate analysis are included in multivariate analysis, some variables not being significant in univariate analysis may appear jointly significant in a multivariate analysis. Thus, in addition to the statistically significant variables related to poor prognosis on the univariate analysis, those also selected by the features selection procedure were also included in the multivariate model. In proteomics studies, the number of samples is usually low compared to the number of variables, due to the limited availability or the cost of measurements. Taking this into account and in order not to introduce bias due to the small sample problem, a wrapper was used as a feature selection method using conditional forward stepwise algorithm based on likelihood rate to reduce the dimensionality of the data [30]. To assess the performance of the multivariate survival model, a leave-one-out cross-validation (LOOCV) analysis was performed. All estimated models using the different training sets in the LOOCV displayed an average goodness of fit (*R*-squared measurement) of 0.914. These results represent that a 91.4% of the variability in the survival time is accounted for by the statistical model. In the test set, this validation showed an accuracy of 92.3%, sensitivity of 85.57% (true-poor prognosis rate) and specificity of 100% (true-fair prognosis rate) for all the left out samples (test samples). All estimated models are depicted in Supplementary Material (Supplementary Figure 1).

The best combination of cytokines selected by the multivariate Cox's proportional hazard analysis is shown in Table 3. None of the clinicopathologic parameters demonstrated a significant trend towards shortened overall survival (*P* > 0.05) and were not considered in the global model. Concerning cytokines at multivariate analysis, expression levels of B7-1/CD80 (*P* = 0.043, HR 77.574, 95% CI (1.138–5289.4)); EG-VEGF/PK1 (*P* = 0.049, HR 1.003, 95% CI (1.000–1.005)) and IL-29 (*P* = 0.026, HR 1.084, 95% CI (1.010–1.164)) showed a significant influence on prognosis. The significant influence on survival observed in univariate analyses for IL-29 was confirmed in multivariate analyses. Beta-coefficients (*β*), hazard ratio (HR), 95% CI, and *P* values for the selected cytokines are shown in Table 3. Although NRG1-beta1/HRG1-beta1 ((*P* = 0.129), HR 1.020, 95% CI (0.994–1.047)) and PD-ECGF ((*P* = 0.108) HR 1.302, 95% CI (0.944–1.797)) failed to significantly influence the prognosis as independent factor, the Cox's proportional hazard analyses using conditional forward stepwise algorithm based on likelihood rate did select them as significant variables that influence the overall survival model (see below).

### 3.4. Prognosis Indexes of Serum Cytokines in PDAC Patients

As combinations of biomarkers are likely to provide more accurate prognosis information, the most accurate subset of variables was sought using the conditional forward stepwise regression approach based on likelihood rate. To illustrate the interrelated effect on OS of the seven markers highlighted by the univariate analysis, the Cox's proportional hazard analysis was employed to select those variables jointly correlated with the survival. As a result of this analysis, a model containing only IL-24 (*P* = 0.026, HR 1.042, 95% CI (1.003–1.023)) and IL-29 (*P* = 0.0.017, HR 1.014, 95% CI (1.005–1.081)) was returned. The overall model fit was shown to be significant by the chi-squared statistic test (*P* = 0.0023). So as to establish a prognosis index to determine PDAC patients overall survival, these cytokines *β*-coefficients were entered in the Cox's model [27] and the following PI model was generated:

(1) $$\begin{array}{c} {\text{PI}}_{\text{univariate}}=0.042\times \text{IL-}24+0.014\times \text{IL-}29. \end{array}$$

Note that PI_univariate_ represents the multivariate model derived from the combination of the underlined markers in the univariate analysis.

Regarding filtered cytokines obtained by multivariate analysis, a second statistically significant (*P* = 0.0003) survival model was built and the following PI model was generated:

(2) PImultivariate=4.351×B7-1CD80+0.003×EG-VEGFPK1+0.081 ×IL-29+0.020×NGR1-beta1HRG1-beta1 +0.264×PD-ECGF.

Note that PI_multivariate_ represents the multivariate model derived from the best of all possible combinations using the cytokines in the multivariate analysis having being previously selected by the wrapper feature selection method.

**Figure 2:**
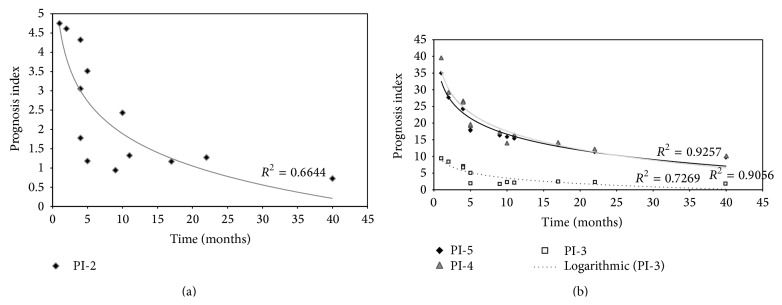
The Cox's regression model. Observed (denoted by square, diamonds and triangles points) and predicted (denoted by solid line) prognosis curves for the PDAC patients according to (a) univariate o and (b) multivariate Cox's proportional hazard model analysis. As explained in the text, the stepwise procedure based on the likelihood ratio was used to select a model containing a statistically significant subset of prognosis factors. The three predicted prognosis curves in (b) are derived from step 3 (where three cytokines are included), step 4 (four cytokines included), and step 5 (five cytokines included) of this stepwise procedure. The predicted survival curves are adjusted to a logarithmic distribution function, as expected. The coefficient of determination* R* ^2^ is illustrative of the model goodness of fit. As coefficient attested, these models would yield useful predictions, being the five cytokines multivariate model the most accurate, reaching a 92.6%. This means that our PI properly models approximately 93% of the survival variation.

Whether these PI can contribute to accurately model survival for this patient cohort was assessed by regression analyses.* R*-squared measurement was given as a proof of goodness of fit. Applying the equations for both PIs, scores of the proposed PI_univariate_ and PI_multivariate_ were calculated, ranked, and correlated to OS. As expected, both survival models showed a logarithmic tendency when plotted against time.  Figure 2 depicts observed PI scores and predicted logarithmic adjustments for these models. For the multivariate model, although the overall model with five cytokines was probed to be statistically significant, regression analyses for models containing 3 and 4 cytokines were also evaluated.* R*-squared values obtained were 0.664, 0.727, 0.906, and 0.926 for PI_univariate_ and 3, 4, and 5 cytokines PI_multivariate_, respectively. These results can be translated into that 66.4%, 72.7%, 90.6%, and 92.6%, respectively, of the variability in the survival time are accounted for by the statistical model. All models yield satisfactory results but multivariate model embracing B7-1/CD80, EG-VEGF/PK1, IL-29, NRG1-beta1/HRG1-beta1, and PD-ECGF stood out from the rest.

**Figure 3:**
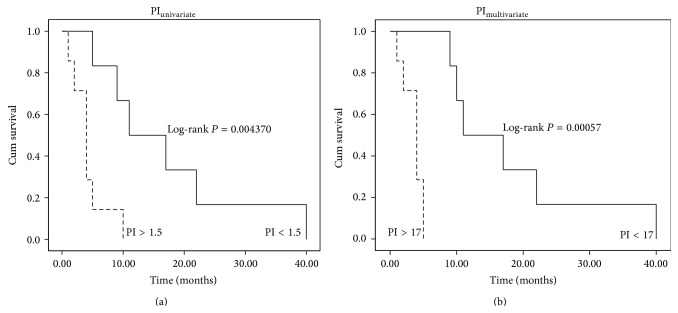
Kaplan-Meier PI survival curves. (a) shows survival plot for PI derived from univariate model, embracing 2 cytokines. A cut-off of 1.5 was chosen to divide cohort of patients in short (<6 months) and long (>6 months) survival times. (b) shows survival plot for PI derived from multivariate model, embracing 5 cytokines. A cut-off of 17 was chosen to divide cohort of patients in short (<6 months) and long (>6 months) survival times. Both PI cut-off values were established considering the best discrimination between poor and fair prognosis. The *P* values for the log-rank tests are shown for both comparisons.

Prognosis index for multivariate model with these five cytokines ranged from 0 to 40 in our cohort. Patients were categorized into two groups according to their prognosis index: poor prognosis (PI > 17) and fair prognosis (PI < 17). Survival curves were then compared among these two prognosis groups (Figure 3(b)). The proposed groups are able to properly differentiate between low (<6 months) and high (>6 months) overall survival time. Overall survival in these groups was highly statistically significant (*P* < 0.00056). Indeed, as shown in Supplementary Table 2, the 100% of the PDAC patients were correctly classified as long/short survival according to the previously proposed cut-off in the prognosis index (PI = 17). Prognosis index for univariate model was also depicted and it ranged from 0 to 5. According to this PI, patients were again categorized into two groups: poor (PI > 1.5) and fair prognosis (PI < 1.5). Furthermore, survival curves were compared among these two prognosis groups (Figure 3(a)) and a significant correlation with the overall survival was also obtained as low (<6 months) and high (>6 months) survival. Overall survival in these groups was less but still significant (*P* < 0.004) compared with the PI multivariate.

## 4. Discussion

In this work, we have conducted an extensive analysis of serum prognosis biomarkers using an antibody array comprising 507 human proteins including cytokines, chemokines, adipokines, growth factors, angiogenic factors, proteases, soluble receptors, soluble adhesion molecules, and other proteins. The main objective of this analysis was to determine if a specific cytokine panel in patient before Gemcitabine and Erlotinib treatment could influence the survival time after this treatment. This is a powerful tool with great potential in applications for biomarker discovery [33]. To assess the impact of altered cytokine profiles on overall survival (OS), Cox's proportional hazard modeling and Kaplan-Meier curves were developed. The effect of serum cytokines levels on OS was dually explored. Initially, a univariate analysis of the cytokines along with some clinicopathologic features was carried out to determine possible significant explanatory variables to model a prognosis index (PI). Whilst univariate analysis returned those highly significant markers to be used as independent prognosis factors, it must not be implied that the combination of these markers would represent the best performance for the multivariate model. Furthermore, univariate selection methods have certain restrictions and may lead to less accurate classifiers. Hence, the most adequate approach to define the multivariate model would be independent from the former, so disregarded variables could also be considered to complete the multivariate model. Then, as some variables may not be significant in univariate analysis but become significant in multivariate analysis, a multivariate approach was used in this study to identify jointly measurable factors that could be used to model risk of PDAC mortality. To overcome the noise and overfitting problem derived from the fact that there were more candidate features than samples, a robust feature selection model was carried out [34]. As long as feature selection is performed reasonably, accurate prediction is achieved even with the simplest of the predictive models [35].

In the course of our evaluation, we first identified 2 cytokines that correlated with patients' prognosis in univariate analysis. Following, a panel of 5 cytokines clearly demonstrated a remarkably better overall performance for modeling OS. Therefore, the multivariate model consisting of B7-1/CD80, EG-VEGF/PK1, IL-29, NRG1-beta1/HRG1-beta1, and PD-ECGF was shown to be more accurate than the univariate model considering the most significant markers. The effectiveness of our model is clearly demonstrated with the evaluation performed by the LOOCV.

Notwithstanding proposed roles for B7-1/CD80, EG-VEGF/PK1, and NRG1-beta1/HRG1-beta1 in PDAC, to the best of our knowledge this is the first time that this combination of serum cytokines has been collectively described as prognosis factors for PDAC. An overview of these biomarkers is subsequently given.

*B7-1/CD80*. The B7 system is one of the most important secondary signaling mechanisms and is essential in maintaining the delicate balance between immune potency and suppression of autoimmunity. B7-1 (CD80) and B7-2 (CD86) are ligands expressed on antigen-presenting cells and they are responsible of the costimulatory signaling whereby T cell priming, growth, maturation, and tolerance are regulated. Upon binding to their receptors, T cell activation and survival are promoted. On the other hand, they can also deliver coinhibitory signaling binding to their inhibitory receptors and blocking T cell response [36]. An inadequate costimulation has been suggested to contribute to the progressive growth of tumours [37]. The combination of B7-1 and B7-H1 has been proposed as prognosis factor for PDAC [38, 39]. Although the role of B7-1 seems to be antitumoral, overall emerging picture is that the aberrant or unbalanced expression of B7 family members can contribute to the escape of the immune surveillance.

*EG-VEGF/PK1*. This molecule was first described as an example of a class of highly specific mitogen that acts to regulate proliferation and differentiation of the vascular endothelium in a tissue-specific manner. Although this protein does not show any structural homology to the VEGF family, they do share multiple regulatory functions related to proliferation and migration [40].* EG-VEGF/PK1* has been described to be related to multiple cancer types including ovarian [41], colorectal [42], prostate [43], hepatic [44], pancreatic [45] and neuroblastoma [46]. It has also been described as a factor for placenta angiogenesis [47].

*IL-29*. Also referred as IFN-*λ*1, it belongs to the type III INF family and it has been described to induce similar biological activities to type I INF family (INT-*α* and *β*). Although both are able to induce antiproliferative responses in many cell types, IFN-*λ*1 appears to be more limited. Signalling via the IFN-*λ*1 results in activation of STATs, MAPKs and PI3K pathways [48–50]. However, the ability of IFN-*λ*1 to trigger these alternative pathways could be cell-type specific or altered in cancer cells. Contrasting conclusion has been derived from other study that suggests growth induction in human multiple myeloma cells through MAPK activation [51]. The precise role of IL-29 in the host responses and immune surveillance has yet to be defined in the context of cancer in general and in PDAC in particular.

*NRG1-beta1/HRG1-beta1*. Neuregulin-1 or heregulin-1 is an extracellular protein ligand meant to bind to the ErbB receptors family members, ErbB3 and ErbB4. Upon interaction with their receptor, a wide range of biological events including the induction and progression of several epithelial cancers are prompted. The NRG1/HRG1 proteins play essential roles in the nervous system, heart, and breast and are involved in the development of human diseases, including schizophrenia and breast cancer [52, 53]. An upregulation of the angiogenic factor VEGF by NRG1/HRG1 has also been described [54]. Their proliferative effects are likely to be achieved through the combined action of multiple pathways, including PI3K, MAPK, and p38MAPK pathways [55] which has been specifically described in PDAC cells. A worse survival rate was related to those PDAC patients with higher expression of HRG-*β* mRNA [56]. ErbB3 has a pivotal role in pancreatic tumorigenesis promoting in vitro and in vivo cancer cell proliferation [57]. It has been recently described that cancer-associated fibroblasts release NRG1/HRG1 ligand, activating PDAC cells by ErbB3/AKT-mediated signalling and enhancing tumorigenesis. This could be related to the insufficient effect of Erlotinib (EGFR inhibitor) when combined with Gemcitabina in PDAC patient treatment [58].

*PD-ECGF*. It is also known as thymidine phosphorylase; its activity and expression in carcinomas of the esophagus, stomach, colorectum, pancreas, and lung are significantly higher than in the adjacent nonneoplastic tissue and may have an important role in the proliferation of these solid tumours. PD-ECGF is expressed not only in the tumor cells but also in the tumor associated stromal cells [59, 60]. Regression analyses in bladder, colorectal, gastric, renal and pancreatic carcinoma have marked PD-ECGF as a prognosis factor for poor outcome [61].

It may not be possible for one single biomarker to provide the necessary prognosis information about the patient to base treatment options on. For this reason, panels of biomarkers are advisable to accurately predict the stage of the disease and how it will progress. Previous studies have indicated that tumor prognosis is closely associated with immune escape by tumor cells. A dynamic relationship between the host immune system and cancer is emerging [62]. Present prognosis scoring system, based on serum cytokines, has been developed to identify patients at the highest risk of cancer progression and death. Due to the emerging role of tumour microenvironment on cancer progression and aggressiveness, cytokines could represent successfully predictors of cancer outcomes as they can be considered as a reflection of the complex tumour immunosuppressive network underlying PDAC. The worsened prognosis associated with tumors harboring this cytokine panel could be associated to a deregulation of growth factor-mediated paracrine loops, particularly in relation to proliferation and angiogenesis. Given the interplay between B7-1/CD80, EG-VEGF/PK1, IL-29, NRG1-beta1/HRG1-beta1, and PD-ECGF and poor prognosis, these cytokines could be considered as novel molecular targets that may lead to more successful therapeutic modalities for PDAC patients.

We are aware of the limitation imposed by population size in this study. However, we have tried to apply a robust statistical analysis and validation. Although PDAC is amongst the less prevalent cancer and studies with large sample size are difficult to be carried out, its aggressiveness and the poor outcome urge to search novel prognosis biomarkers as the basis for rational treatment decisions, analysis of novel therapeutic interventions, and tailored treatment approaches [63]. For this model to be applied in clinical decisions making, further validations are imperative in order to assure that this combination of cytokines would successfully model the outcome in other patients populations.

In summary, we have identified for the first time a panel of five serum cytokines comprising B7-1/CD80, EG-VEGF/PK1, IL-29, NRG1-beta1/HRG1-beta1, and PD-ECGF with prognosis significance in PDAC. These molecules might not only allow a more accurate prediction of prognosis of patients with PDAC but also represent novel targets for therapeutic agents. Studies in prognosis biomarkers achieving true clinical impact and improving patient management and outcome are a matter of the utmost importance in PDAC. Besides, being able to foresee the prognosis of a PDAC patient may help to avoid futile therapy approaches and to improve quality of life of those whose long-term survival is unpromising.

## Supplementary Material

Supplementary Figure 1 shows all estimated models using the different training sets in the Leave-one-out cross-validation (LOOCV). LOOCV was applied as a useful technique for assessing the performance of the model. One sample is removed from the initial 14 sample dataset, leaving a temporary 13 sample training set and one left out-sample. The R-squared measurement indicates the models goodness of fit. Supplementary Table 1 provides the clinicopathological data of the 14 PDAC patients including their overall survival. Supplementary Table 2 compares the real and estimated overall survival for the 14 PDAC patients. Finally, supplementary Table 3 lists the whole normalized data of the cytokine antibody array.

## Figures and Tables

**Table 1:** Clinicopathologic characteristics of the study population (*n* = 14).

Age at diagnosis, years (mean ± StD)	66 ± 10.5

Gender	Male: 50% Female: 50%

Disease stage	III (28%) IV (72%)

Type of chemotherapy	Gemcitabine + Erlotinib

Clinical response	PR (14.29%) SD (21.43%) PD (64.28%

Survival time, months (mean ± StD)	12.6 ± 12.6

Outcome:	
Follow-up months (mean ± StD)	12.6 ± 12.6
Death from pancreatic cancer	100%
Alive	0%
Lost to follow-up (censored cases)	0%

CEA level [*μ*g/L] (mean ± StD)	2219 ± 5017
	Healthy: 0–37

CA 19-9 level [U/L] (mean ± StD)	899 ± 3185
	Healthy: 0–5

PR: partial response; SD: stable disease; PD: progressive disease; StD: standard deviation.

**Table 2:** Prognosis factors in univariate analysis.

Variable	Overall survival				
	*β*	HR	95% CI		*P* value
BDNF	0.005	1.005	1.000	1.009	0.034
HVEM/TNFRSF14	−0.079	0.924	0.858	0.996	0.038
IL-24	0.040	1.041	1.006	1.078	0.023
IL-29	0.012	1.012	1.002	1.023	0.021
Leptin R	0.008	1.008	1.001	1.015	0.018
LRP-6	0.027	1.027	1.004	1.051	0.022
ROBO4	0.002	1.002	1.000	1.004	0.045
Age	0.086	1.089	1.008	1.177	0.030
Clinical response	2.064	8.706	1.057	71.692	0.013

Cytokines	Overall model fit (*P* = 0.0023)				
	*β*	HR	95% CI		*P* value

IL-24 (1)	0.042	1.042	1.003	1.023	0.026
IL-29 (2)	0.014	1.014	1.005	1.081	0.017

*β*: coefficient provided by the Cox's regression model for a particular patient and cytokine; HR: hazard ratio (represents the factor by which the hazard changes for each one-unit increase of the cytokine expression); 95% CI: upper and lower limits of the confidence interval with a significance level of 0.05.

**Table 3:** Prognosis factors in multivariate analysis.

Cytokines	Overall survival					Overall model fit
	*β*	HR	95% CI		*P* value	*P* value
IL-29	0.081	1.084	1.010	1.164	0.026	0.004212
B7-1/CD80	4.351	77.574	1.138	5289.45	0.043	0.002494
PD-ECGF	0.264	1.302	0.944	1.797	0.108	0.001350
EG-VEGF/PK1	0.003	1.003	1.000	1.005	0.049	0.000134
NRG1-beta1/HRG1-beta1	0.020	1.020	0.994	1.047	0.129	0.000286

Cytokines	Overall survival in the univariate analysis					
	*β*	HR	95% CI			*P* value

IL-29	0.012	1.012	1.002		1.023	0.021
B7-1/CD80	0.373	1.452	0.876		2.407	0.148
PD-ECGF	0.044	1.045	0.997		1.096	0.068
EG-VEGF/PK1	−0.0001	1.000	0.999		1.000	0.640
NRG1-beta1/HRG1-beta1	−0.004	0.996	0.979		1.014	0.673

*β*: coefficient provided by the Cox's regression model for a particular patient and cytokine; HR: hazard ratio (represents the factor by which the hazard changes for each one-unit increase of the cytokine expression); 95% CI: upper and lower limits of the confidence interval with a significance level of 0.05.

## Abbreviations

PDAC: Pancreatic ductal adenocarcinoma
OS: Overall survival
HR: Hazard ratio
PI: Prognosis index
CI: Confidence interval.
