# Supplementary material for: Prognosis Relevance of Serum Cytokines in Pancreatic Cancer
Source: Biomed Res Int. 2015 Aug 4;2015:518284. doi: 10.1155/2015/518284 (PMC4539422; doi:10.1155/2015/518284)
Supplement: Supplementary file 1 — Supplementary Figure 1 shows all estimated models using the different training sets in the Leave-one-out cross-validation (LOOCV). LOOCV was applied as a useful technique for assessing the performance of the model. One sample is removed from the initial 14 sample dataset, leaving a temporary 13 sample training set and one left out-sample. The R-squared measurement indicates the models goodness of fit. Supplementary Table 1 provides the clinicopathological data of the 14 PDAC patients including their overall survival. Supplementary Table 2 compares the real and estimated overall survival for the 14 PDAC patients. Finally, supplementary Table 3 lists the whole normalized data of the cytokine antibody array. [file 518284.f1.pdf]

Supplementary material:

Supplementary Figure 1: Leave-one-out cross-validation (LOOCV) analysis. A LOOCV was performed to estimate the predictive value of our five cytokines multivariate Cox model. For this analysis, a model was constructed using every sample (the training set) except one (the test set). The entire procedure was repeated until each case had been omitted once. In every iteration, one sample was left out and the multivariate Cox model was constructed by the rest of the samples using exactly the same process as our modeling procedure for feature selection and model construction. Subsequently, the test sample PI was obtained from the previously performed model. All constructed models are shown with their respective goodness of fit (R-squared measurements) ranging from 0.8 to 0.98.

Supplementary Table 1: Individualized clinicopathologic data of the 14 PDAC patients and their classification according to long and short survival.

Supplementary Table 2: Relation between the real and the estimated survival based on multivariate prognosis index for the 14 PDAC patients. The cytokine expression levels for the 5 cytokines involved in the multivariate prognosis model are shown. Long and short survivals are estimated from PI values taking into account the cut-off provided in Figure 3 (PI<17 for long survival and PI>17 for short survival).

Supplementary Table 3: Normalized data from antibody array for 507 human cytokines and 14 PDAC patients.

Supplementary Figure 1:

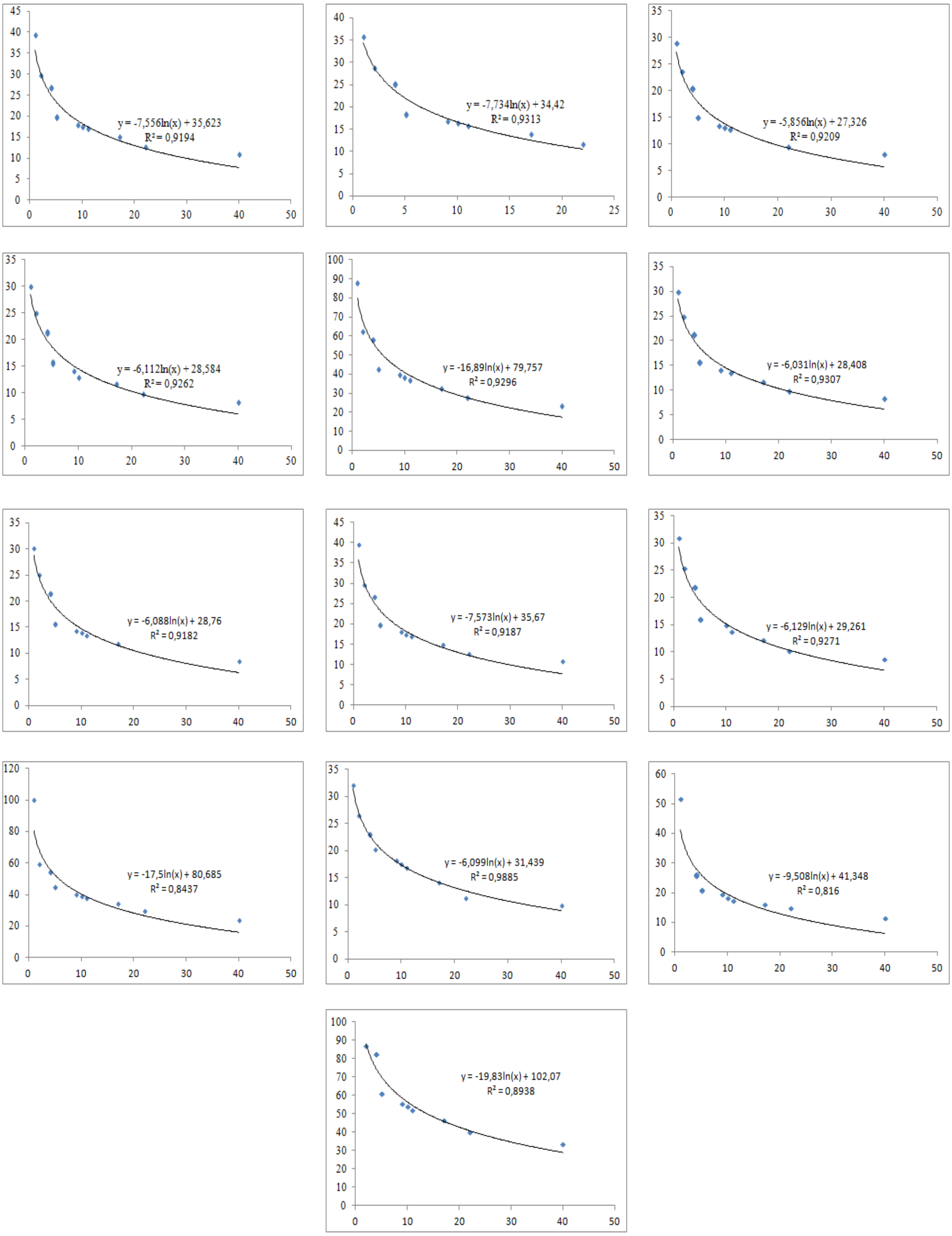

Supplementary Table 1:

Supplementary Table 1: Individualized clinicopathologic data.									
	Age	***Gender***	***Disease Status***	***Diabetes***	***Smoke***	***Chemotherapy***	***Clinical Response***	***Survival time***	***Long/Short Survival***
P1	67	Male	III	Type II	Yes	Gem+Erl	PD	4	S
P2	41	Male	IV	No	No	Gem+Erl	SD	40	L
P3	62	Male	IV	No	No	Gem+Erl	PR	17	L
P4	72	Female	IV	Type II	No	Gem+Erl	PD	11	L
P5	55	Female	IV	No	No	Gem+Erl	PD	5	S
P6	78	Female	III	No	No	Gem+Erl	SD	37	L
P7	57	Male	IV	Type II	Yes	Gem+Erl	PD	15	L
P8	60	Male	IV	No	Yes	Gem+Erl	PR	22	L
P9	79	Male	IV	No	No	Gem+Erl	PD	4	S
P10	71	Female	III	No	No	Gem+Erl	PD	9	L
P11	78	Female	III	No	No	Gem+Erl	PD	4	S
P12	61	Female	IV	Type II	No	Gem+Erl	SD	5	S
P13	70	Male	IV	Type II	Yes	Gem+Erl	PD	2	S
P14	70	Female	IV	Type II	No	Gem+Erl	PD	1	S
PR: partial response; SD: stable disease; PD: progressive disease; S: short survival; L: long survival									

Supplementary Table 2:

Supplementary Table 2:Real vs Estimated survival based on multivariate prognosis index										
	**Real Survival**		**Cytokine Levels**					**Estimated Prognosis**		**Goal**
	**Survival time**	**Long / Short**	**B7-1/CD80**	**IL-29**	**PD-ECGF**	**EG-VEGF/PK1**	**NRG1-beta1 / HRG1-beta1**	**Estimated PI**	**Estimated survival**	
**P1**	4	S	4.55	27.28	1.24	632.39	19.43	24.37	S	✓
**P2**	40	L	1.21	2.82	5.23	869.68	28.16	9.71	L	✓
**P3**	17	L	0.75	30.14	11.05	1643.14	30.65	13.53	L	✓
**P4**	11	L	0.81	33.22	4.05	3033.63	9.18	15.39	L	✓
**P5**	5	S	0.91	22.77	2.73	4348.38	5.77	18.00	S	✓
**P6**	37	L	1.26	1.26	6.74	2690.59	1.26	14.44	L	✓
**P7**	15	L	1.08	9.4	10.12	1730.97	160.81	15.87	L	✓
**P8**	22	L	0.89	12.82	14.31	846.04	24.45	11.39	L	✓
**P9**	4	S	4.51	20.05	5.51	308.34	36.1	24.23	S	✓
**P10**	9	L	1.28	1.28	1.28	3770.85	21.78	16.30	L	✓
**P11**	4	S	1.22	137.51	26.53	279.89	9.76	24.36	S	✓
**P12**	5	S	1.27	73.79	23.19	14.34	5.9	17.77	S	✓
**P13**	2	S	1.05	277.23	1.05	142.83	2.81	27.68	S	✓
**P14**	1	S	1.52	60.8	86.04	169.34	13.98	34.99	S	✓
S: short survival; L: long survival; PI: prognosis index										

Supplementary Table 3:

Cytokine Name	P1	P2	P3	P4	P5	P6	P7	P8	P9	P10	P11	P12	P13	P14
**6Ckine**	22.73	20.52	0.75	5.13	0.91	1.26	1.08	9.54	1.50	1.28	184.15	159.81	7.72	126.77
**Activin A**	31.83	37.41	0.75	0.81	0.91	1.26	1.08	11.93	1.50	1.28	28.66	8.43	1.05	13.98
**Activin B**	4.55	39.02	0.75	0.81	0.91	1.26	1.08	22.96	186.51	1.28	31.40	11.81	3.16	19.15
**Activin C**	2.89	25.74	8.79	0.81	0.91	1.26	1.08	16.70	1.50	1.28	7.62	4.22	7.72	13.38
**Activin RIA / ALK-2**	1.65	26.95	10.30	1.89	0.91	1.26	1.08	30.12	31.59	2.14	59.45	124.39	3.51	54.72
**Activin RIB / ALK-4**	12.81	15.29	12.06	9.72	31.27	16.84	1.08	17.59	268.73	16.65	16.46	7.17	1.05	7.30
**Activin RII A/B**	14.47	16.49	11.81	9.99	36.43	13.47	1.08	7.16	21.06	14.52	7.62	8.01	1.05	7.60
**Activin RIIA**	269.49	276.35	356.21	300.07	261.68	332.22	81.31	115.41	311.85	182.35	26.53	188.06	435.50	80.56
**Adiponectin / Acrp30**	390.18	113.03	15.32	100.47	68.30	123.37	53.12	268.40	280.26	251.11	154.89	88.55	23.86	200.04
**AgRP**	14.47	18.50	2.51	13.23	10.02	1.26	1.08	13.42	514.39	14.09	86.89	17.29	1.05	16.11
**ALCAM**	9.09	22.93	0.75	14.58	11.54	1.26	1.08	5.96	22.06	17.94	3.05	1.27	1.05	3.34
**Angiogenin**	18.60	21.72	18.84	19.99	17.30	7.16	1.08	21.77	24.07	14.09	40.55	4.22	1.05	24.32
**Angiopoietin-1**	45.05	28.56	34.67	34.84	29.14	2.11	1.08	39.36	104.28	37.58	178.06	107.95	50.88	114.92
**Angiopoietin-2**	69.44	50.68	132.64	105.33	131.75	1.26	16.62	51.00	157.93	73.03	2200.10	1749.50	364.26	1883.68
**Angiopoietin-4**	35.13	32.18	49.74	0.81	0.91	1.26	78.06	8.65	21.56	7.26	41.16	12.65	1.05	29.49
**Angiopoietin-like 1**	2685.80	2937.27	2502.51	4582.86	0.91	3142.81	2081.50	0.89	5800.22	2956.04	3631.87	3017.46	2587.71	2900.62
**Angiopoietin-like 2**	40.09	29.36	117.31	0.81	0.91	8.42	48.42	0.89	1.50	1.28	117.99	77.17	11.58	91.20
**Angiopoietin-like Factor**	22.73	1.21	0.75	0.81	0.91	1.26	16.98	0.89	1.50	2.14	15.85	10.54	1.05	12.46
**Angiostatin**	5276.54	1.21	0.75	748.41	0.91	668.65	1770.00	314.62	1.50	1.28	3358.99	2639.22	1431.07	3134.10
**APJ**	5.79	1.21	0.75	0.81	2.13	2984.92	1.08	1446.95	10331.00	1926.42	0.91	1.27	1.05	5.47
**AR (Amphiregulin)**	1.24	25.34	10.80	0.81	16.09	1.26	57.82	7.16	8.52	14.95	25.92	4.64	1.05	11.55
**APRIL**	1.24	1177.80	1241.46	1432.28	718.25	520.01	432.56	339.67	5596.67	1621.08	542.71	356.31	1206.83	212.81
**Artemin**	1.24	40.63	0.75	0.81	0.91	1.26	86.37	36.38	12.53	81.99	16.77	2.53	1.05	11.86
**Axl**	5.37	77.64	0.75	0.81	0.91	1.26	1.08	0.89	1.50	1.28	14.94	1.27	1.05	8.21

**B7-1 /CD80**	4.55	1.21	0.75	0.81	0.91	1.26	1.08	0.89	4.51	1.28	1.22	1.27	1.05	1.52
**BAFF R / TNFRSF13C**	46.71	20.52	0.75	0.81	0.91	7.16	1.08	9.24	1.50	1.28	9.45	9.28	1.05	16.11
**BCMA / TNFRSF17**	38.44	20.11	11.05	11.07	16.39	21.90	1.08	22.96	4.01	14.09	5.49	5.90	1.05	2.74
**BD-1**	39.68	16.89	18.34	19.72	11.23	21.05	1.08	36.08	36.10	22.63	28.05	13.49	8.07	16.42
**BDNF**	52.08	53.90	72.10	46.46	69.52	58.95	6.50	54.28	89.74	74.73	193.30	291.79	628.15	124.95
**beta-Catenin**	1.24	7.24	5.02	8.37	3.95	1.26	1.08	19.38	1.50	1.28	1.83	1.27	1.05	1.22
**beta-Defensin 2**	1.24	16.49	11.05	0.81	0.91	1.26	1.08	34.00	1.50	1.28	1.22	1.27	1.05	0.91
**beta-NGF**	1.24	43.44	12.06	0.81	0.91	1.26	1.08	31.01	6.02	1.28	41.16	36.26	1.40	27.97
**BIK**	1.24	15.29	0.75	0.81	0.91	1.26	1.08	9.54	20.05	1.28	1.83	1.27	1.05	0.91
**BLC / BCA-1 / CXCL13**	1.24	30.97	12.31	0.81	3.34	1.26	1.08	1.79	63.17	2.14	17.99	22.77	1.05	18.54
**BMP-2**	1.24	13.68	0.75	0.81	0.91	1.26	1.08	3.88	14.04	2.56	2.74	5.06	2.46	4.56
**BMP-3**	28.52	28.16	10.80	2.70	1.82	1.26	1.08	30.72	116.32	7.26	21.04	14.76	11.93	10.94
**BMP-3b / GDF-10**	28.93	15.29	0.75	0.81	3.04	1.26	1.08	20.28	17.55	1.28	24.09	39.22	1.05	17.33
**BMP-4**	28.52	39.42	0.75	0.81	5.46	1.26	4.34	44.73	112.30	11.10	10.98	15.18	6.32	14.90
**BMP-5**	3.31	35.00	24.12	0.81	0.91	1.26	1.08	14.02	22.56	1.28	35.67	8.43	1.05	22.19
**BMP-6**	1.24	1.21	6.53	0.81	3.04	2.11	1.08	0.89	1.50	1.28	6.10	1.27	2.11	4.26
**BMP-7**	1.24	26.95	13.82	2.43	6.38	1.26	1.08	5.07	3.01	1.28	13.72	1.27	1.05	4.86
**BMP-8**	62.83	40.23	18.34	12.69	7.29	2.53	1.08	70.38	385.54	98.22	19.51	1.27	6.67	11.86
**BMP-15**	42.99	13.68	3.27	2.70	0.91	2.53	1.08	17.59	1.50	20.50	31.71	1.27	1.05	15.50
**BMPR-IA / ALK-3**	25.21	13.27	5.28	3.51	2.13	5.47	1.08	17.30	2.51	15.37	25.00	1.27	1.05	15.50
**BMPR-IB / ALK-6**	234.36	102.98	95.21	100.47	159.07	152.00	163.34	148.81	788.64	364.27	292.09	219.27	219.68	155.66
**BMPR-II**	20.25	14.48	14.07	11.34	17.30	24.00	63.60	13.72	36.10	12.81	100.00	67.05	3.51	61.41
**BTC**	10.33	37.81	11.81	10.80	18.21	22.74	48.79	16.70	18.05	4.27	149.40	99.09	4.91	168.42
**Cardiotrophin-1 / CT-1**	2.48	32.18	13.06	3.78	0.91	1.26	1.08	1.79	4.01	2.14	90.25	89.82	1.05	67.49
**CCL14 / HCC-1 / HCC-3**	1.24	27.76	3.01	0.81	0.91	1.26	1.08	0.89	1.50	2.14	100.61	35.00	1.05	66.28
**CCL28 / VIC**	1.24	30.57	3.77	2.97	1.52	5.47	1.08	11.93	1.50	1.28	60.37	42.59	1.05	46.51
**CCR1**	27.69	1.21	10.05	0.81	0.91	1.26	1.08	45.93	195.53	1.71	91.16	44.70	3.86	35.87

**CCR2**	10.33	5.23	5.02	2.16	0.91	2.11	1.08	5.96	35.60	1.28	42.38	49.76	6.67	28.88
**CCR3**	11.16	4.42	2.26	3.24	0.91	5.89	1.08	45.03	48.63	1.28	28.05	5.48	1.05	11.55
**CCR4**	39.27	24.54	13.31	2.97	6.38	8.42	1.08	0.89	283.27	5.55	19.21	15.18	13.69	17.02
**CCR5**	12.40	28.96	13.31	1.35	6.68	1.26	1.08	2.68	13.04	1.28	14.94	15.18	2.11	9.42
**CCR6**	19.43	12.07	9.80	0.81	7.59	1.26	1.08	2.68	21.06	1.28	7.62	7.59	5.97	7.60
**CCR7**	1388.78	1513.28	405.70	169.35	286.57	554.54	622.64	691.27	582.58	257.94	84.46	358.42	808.18	179.07
**CCR8**	100.85	84.07	22.86	17.02	85.00	29.47	1.08	51.29	46.63	26.05	14.63	54.40	86.68	31.92
**CCR9**	390.59	808.94	202.47	55.10	237.09	268.64	185.38	201.30	449.22	92.67	40.25	296.85	404.97	147.75
**CD14**	1.24	15.69	6.28	13.50	36.13	1.26	1.08	2.09	9.53	1.28	11.28	8.86	1.40	6.99
**CD27 / TNFRSF7**	1.24	1.21	2.51	15.40	15.18	1.26	1.08	6.26	66.68	4.27	68.91	25.72	1.05	21.89
**CD30 / TNFRSF8**	1.24	1.61	2.01	3.78	11.84	1.26	1.08	0.89	53.14	1.28	2.74	1.69	1.05	1.82
**CD30 Ligand / TNFSF8**	16.95	17.70	10.55	10.80	32.48	4.63	1.08	32.80	67.18	21.35	6.10	1.27	1.05	4.56
**CD40 / TNFRSF5**	14.88	5.63	8.29	9.45	10.32	14.32	1.08	4.18	108.79	12.38	19.51	5.06	1.05	11.86
**CD40 Ligand / TNFSF5 /CD154**	125.24	179.81	66.82	55.10	55.86	94.74	17.71	60.54	139.88	41.42	86.89	25.30	38.25	56.85
**CD 163**	1.24	4.42	8.79	11.07	3.04	17.26	1.08	3.58	26.57	3.84	18.90	10.54	3.86	14.29
**Cerberus 1**	1.24	1.21	1.26	7.56	3.95	13.47	1.08	3.58	37.10	7.26	2.13	1.27	1.05	0.91
**Chem R23**	1.24	1.21	1.51	35.11	0.91	56.84	1.08	0.89	48.63	2.56	5.49	3.80	2.81	4.56
**Chordin-Like 1**	11.99	13.27	11.56	15.67	0.91	16.00	1.08	0.89	157.43	11.10	75.92	48.07	1.05	22.19
**Chordin-Like 2**	95.48	34.59	62.55	55.10	16.09	68.21	1.08	10.44	56.65	23.91	90.25	137.46	65.97	56.55
**Csk**	11.57	10.86	12.31	10.53	0.91	18.53	1.08	0.89	20.05	16.65	21.34	1.27	1.05	8.51
**CLC**	41.75	60.74	20.10	12.69	21.25	25.26	1.08	65.61	824.23	98.65	28.35	1.27	3.51	18.24
**CNTF**	1011.83	588.90	615.20	388.39	443.82	607.59	295.60	280.32	1923.71	743.49	110.37	204.51	364.61	104.28
**CNTF R alpha**	48.36	20.11	17.33	14.31	0.91	19.37	1.08	28.93	150.91	27.33	23.78	18.13	4.91	16.42
**Coagulation Factor III / Tissue Factor**	112.84	14.08	3.27	2.70	0.91	1.68	1.08	28.63	14.04	1.28	39.03	25.30	1.75	29.79
**CRIM 1**	15.29	27.76	10.55	2.16	0.91	1.68	1.08	16.70	33.09	1.28	98.48	120.60	1.05	72.66
**Cripto-1**	23.97	32.18	2.01	1.35	0.91	1.26	1.08	17.30	26.07	1.71	31.10	20.24	3.16	13.68

**CRTH-2**	78.95	37.81	61.55	71.84	106.55	1.68	155.39	119.88	268.23	183.20	25.31	63.25	140.02	4.56
**Cryptic**	42.16	39.42	38.94	15.67	0.91	1.26	1.08	22.96	20.05	9.40	247.27	271.98	2.81	169.64
**CTACK / CCL27**	177.32	83.27	122.34	56.72	138.43	1.26	68.30	81.12	545.98	319.01	194.52	450.34	462.52	110.36
**CTGF / CCN2**	14.88	33.39	8.04	1.35	0.91	1.26	1.08	5.37	27.07	3.42	78.05	72.95	1.05	44.69
**CTLA-4 /CD152**	25.21	17.30	5.02	3.78	0.91	2.95	1.08	0.89	24.57	1.28	31.10	13.07	8.77	17.63
**CV-2 / Crossveinless-2**	26.87	20.92	3.27	2.97	0.91	2.53	1.08	2.39	18.05	1.28	6.10	4.22	6.32	8.51
**CXCL14 / BRAK**	4546.60	5189.49	1627.31	800.81	1278.65	1392.88	1393.81	3838.06	6477.56	5477.35	1521.11	1672.76	3.51	1679.99
**CXCL16**	33.07	7.64	1.51	7.83	6.98	6.32	1.08	42.05	1.50	1.28	3.35	1.27	2.46	0.91
**CXCR1 / IL-8 RA**	1503.68	650.04	605.91	498.85	602.59	738.97	428.95	859.46	749.53	368.97	117.99	740.87	250.56	341.71
**CXCR2 / IL-8 RB**	4397.39	6146.46	7199.82	6535.06	2945.27	5207.71	3675.16	1163.05	1256.41	501.78	1286.95	1637.34	370.58	646.64
**CXCR3**	47.53	37.41	20.85	10.80	38.55	43.79	1.08	29.52	26.57	22.63	206.11	86.02	1.40	67.19
**CXCR4 (fusin)**	494.34	368.47	330.84	410.80	751.04	828.65	53.84	55.17	140.38	38.01	114.64	146.74	54.39	53.81
**CXCR5 /BLR-1**	16.95	15.29	0.75	11.07	0.91	1.26	1.08	5.67	29.08	1.28	90.86	55.66	2.81	49.86
**CXCR6**	59.93	68.38	145.95	100.20	378.25	126.32	1.08	68.59	112.30	8.97	56.10	46.81	21.06	32.53
**D6**	12.40	19.31	58.78	13.77	0.91	16.84	1.08	16.70	24.57	2.99	121.04	19.82	1.05	56.55
**DAN**	1.24	11.26	0.75	3.78	0.91	1.26	1.08	0.89	49.13	2.99	2.74	1.27	1.75	1.52
**DANCE**	1.24	16.49	2.26	2.43	0.91	1.26	1.08	0.89	12.53	2.99	26.22	1.27	1.05	20.37
**DcR3 / TNFRSF6B**	1.24	38.62	6.53	2.70	0.91	1.26	1.08	5.96	23.56	5.98	183.54	117.65	1.05	103.97
**Decorin**	16.12	89.70	8.54	4.59	0.91	4.63	1.08	0.89	24.07	9.82	60.67	33.73	1.05	40.13
**Dkk-1**	5.37	26.15	0.75	1.62	0.91	1.26	1.08	0.89	550.49	5.12	142.69	98.67	1.05	114.61
**Dkk-3**	3.31	23.33	5.28	1.35	0.91	1.26	1.08	0.89	18.05	4.70	40.86	12.65	1.05	23.11
**Dkk-4**	7.85	16.89	11.30	10.80	1.52	2.53	1.08	4.18	37.10	1.71	67.99	47.65	1.05	24.63
**DR3 / TNFRSF25**	1.24	24.94	33.66	9.99	0.91	1.26	1.08	5.37	7.52	1.28	2.74	1.27	2.11	1.82
**DR6 / TNFRSF21**	1.24	36.61	27.63	29.98	19.43	5.05	1.08	1.19	33.09	1.71	174.70	277.46	2.46	99.41
**Dtk**	17.36	55.11	26.88	5.94	18.52	2.11	1.08	0.89	1046.84	2.56	114.03	27.83	1.05	35.87
**EDA-A2**	23.15	18.91	0.75	0.81	0.91	1.26	1.08	0.89	1.50	2.99	125.92	10.12	1.05	53.20
**EDAR**	97.13	17.30	1.76	0.81	0.91	4.63	1.08	15.81	14.04	1.28	126.84	43.43	1.05	78.44

**EDG-1**	25.63	15.29	3.27	22.96	2.73	1.26	1.08	9.84	2.51	1.28	66.16	47.23	9.83	55.03
**EGF**	12.40	13.27	6.03	0.81	0.91	1.26	1.08	3.28	1.50	1.28	37.20	6.33	1.05	16.11
**EGF R / ErbB1**	49.19	17.30	0.75	18.10	0.91	2.53	1.08	3.28	1.50	5.12	5.49	1.27	1.05	3.65
**EG-VEGF / PK1**	632.39	869.68	1643.14	3033.63	4348.38	2690.59	1730.97	846.04	308.34	3770.85	279.89	14.34	142.83	169.34
**EMAP-II**	114.49	31.38	0.75	8.91	0.91	128.00	1.08	65.61	1.50	17.08	53.97	1.27	1.75	34.35
**ENA-78**	37.20	69.59	5.02	6.21	18.21	21.05	1.08	34.59	109.80	28.19	41.47	32.47	1.05	29.19
**Endocan**	103.33	24.54	2.26	2.97	0.91	1.68	1.08	20.88	16.04	4.27	74.39	36.69	1.05	52.29
**Endoglin / CD105**	24.80	92.92	1.51	0.81	0.91	1.26	1.08	7.46	47.13	1.28	87.50	57.77	1.05	65.36
**Endostatin**	25.21	14.88	0.75	3.24	0.91	4.63	2.89	2.39	30.58	1.28	7.93	1.69	5.26	12.16
**EN-RAGE**	97.96	17.30	9.55	10.80	164.84	2.95	10.84	128.83	13.04	3.84	106.71	50.60	1.05	51.68
**Eotaxin / CCL11**	31.00	80.05	47.48	16.75	44.93	15.16	5.42	57.56	38.60	7.26	67.69	57.77	1.05	44.39
**Eotaxin-2 / MPIF-2**	52.91	53.10	33.91	32.68	27.93	24.00	8.67	31.61	12.53	2.99	85.06	61.99	1.05	49.55
**Eotaxin-3 / CCL26**	4.55	22.93	20.10	33.49	4.55	21.05	32.88	20.88	152.91	7.26	175.62	49.34	2.11	83.00
**Epiregulin**	1.65	21.72	12.06	8.10	0.91	1.26	1.08	16.10	14.54	1.28	56.71	34.16	1.40	23.41
**ErbB2**	2.48	19.31	19.59	10.26	0.91	1.26	2.89	23.86	27.07	1.71	60.67	40.06	12.63	24.32
**ErbB3**	23.56	34.59	85.16	20.53	21.86	26.95	57.10	24.75	37.10	17.94	136.59	84.33	4.56	57.16
**ErbB4**	22.73	22.53	17.58	9.99	26.41	26.53	35.78	26.84	177.98	10.68	62.81	15.18	11.23	21.89
**Erythropoietin**	3700.93	2366.87	5879.48	3163.27	2924.02	2189.11	7505.70	9832.52	10435.78	9943.87	658.87	1507.04	4022.29	419.85
**E-Selectin**	3.72	41.03	25.12	4.32	27.32	1.26	40.11	57.26	294.80	5.98	27.75	15.18	1.05	13.07
**Endothelin**	38.03	29.77	18.84	16.21	64.66	15.58	119.61	25.65	60.66	20.93	24.09	3.80	34.04	13.68
**FADD**	69.85	12.47	0.75	2.43	18.82	1.26	63.96	37.58	19.05	1.28	10.67	1.27	11.58	15.50
**FAM3B**	33.07	13.27	3.77	2.43	0.91	1.26	1.08	2.68	3.51	1.28	64.33	18.55	1.05	29.19
**Fas / TNFRSF6**	16.12	28.96	6.03	4.59	1.82	1.26	1.08	3.28	17.55	1.28	76.53	53.97	5.61	45.91
**Fas Ligand**	28.52	19.31	8.29	5.13	0.91	1.26	3.25	5.37	51.64	1.28	51.53	38.37	1.75	27.36
**FGF Basic**	34.31	20.92	75.36	27.55	21.25	13.05	1.08	19.38	63.17	1.28	82.02	105.00	2.11	64.76
**FGF-BP**	14.05	15.69	23.11	11.34	0.91	1.26	1.08	10.44	16.54	1.28	47.87	20.66	1.05	27.97
**FGF R3**	22.32	49.08	16.08	12.42	0.91	1.26	1.08	7.16	604.14	25.62	120.43	85.60	1.75	91.81

**FGF R4**	6209.83	4879.36	3216.19	3118.44	2212.14	5045.18	4144.22	5800.03	3342.56	6334.86	1332.99	1278.92	1109.62	1164.08
**FGF R5**	44.23	15.69	4.52	0.81	0.91	95.58	37.22	34.59	2.01	1.28	107.32	23.19	1.05	46.21
**FGF-4**	27.69	35.40	19.59	0.81	10.63	24.84	95.04	14.91	20.05	20.07	145.43	110.48	4.56	70.23
**FGF-5**	2.07	31.38	9.80	3.78	6.07	1.26	1.08	9.24	15.54	2.99	23.17	5.90	5.61	11.55
**FGF-6**	1.24	13.27	3.01	2.70	9.71	10.11	1.08	0.89	2.51	1.28	144.82	43.01	1.05	64.76
**FGF-7 / KGF**	1.24	29.77	4.02	1.62	9.11	6.32	1.08	2.98	1.50	1.28	73.48	50.60	1.05	63.84
**FGF-8**	9.09	12.87	11.56	0.81	18.52	1.26	1.08	73.06	14.54	8.97	21.65	2.95	1.05	22.19
**FGF-9**	30.59	20.92	35.42	38.89	26.41	1.26	5.42	25.94	77.21	114.02	569.84	608.89	127.03	672.18
**FGF-10 / KGF-2**	31.41	16.09	0.75	1.35	22.77	1.26	28.19	15.21	39.61	36.30	63.42	11.39	23.16	30.40
**FGF-11**	26.04	15.29	8.04	12.42	2.13	2.95	1.08	28.33	7.02	24.77	12.20	6.75	9.83	9.73
**FGF-12**	97.96	12.87	5.78	11.88	2.43	2.95	1.08	49.21	1.50	3.84	36.59	16.02	1.05	22.50
**FGF-13 1B**	1201.54	862.84	0.75	21.88	3.34	1.26	342.94	697.23	1.50	562.43	23.17	105.00	40.71	21.59
**FGF-16**	34.31	10.46	14.32	7.29	8.20	13.05	24.21	2.98	44.62	12.38	45.43	37.53	5.26	22.19
**FGF-17**	6.20	2.41	10.30	5.40	3.95	1.68	1.08	12.23	5.01	2.14	8.23	2.95	4.56	4.56
**FGF-18**	2.07	2.82	14.32	8.37	7.89	1.26	1.08	16.10	12.03	1.71	11.59	5.90	1.05	7.30
**FGF-19**	200.88	23.33	3.77	6.75	5.16	1.26	15.18	62.03	1.50	5.98	17.07	34.16	12.98	16.42
**FGF-20**	12.40	7.24	4.27	4.59	0.91	2.11	7.59	13.72	1.50	3.84	11.28	8.86	1.05	12.46
**FGF-21**	90.52	4.83	3.01	3.51	0.91	1.68	9.40	43.84	1.50	2.56	18.90	1.27	7.37	8.82
**FGF-23**	1.65	9.65	8.79	5.13	0.91	7.58	38.31	14.61	34.09	9.40	198.18	2.53	3.51	121.61
**FLRG**	3.72	12.07	3.52	2.97	0.91	1.68	17.71	8.35	26.07	2.56	5.49	3.37	1.05	2.74
**Flt-3 Ligand**	5.79	62.75	40.44	8.91	0.91	1.68	5.42	61.13	435.18	2.56	25.92	10.96	1.05	6.38
**Follistatin**	1261.89	953.75	1429.36	372.99	7.59	7.58	118.89	763.44	1055.36	459.08	51.83	226.01	84.92	79.65
**Follistatin-like 1**	74.81	51.49	35.42	34.84	11.54	2.53	100.10	67.40	101.78	53.81	37.81	119.75	66.32	185.75
**Fractalkine**	14.47	16.89	11.05	9.72	2.43	1.26	34.33	18.79	14.54	14.95	7.32	6.75	1.05	6.99
**Frizzled-1**	1.24	4.83	3.52	2.16	3.64	1.26	1.08	0.89	5.51	3.42	14.94	7.17	1.05	6.08
**Frizzled-3**	1.24	1.61	3.27	2.97	0.91	1.26	1.08	1.49	4.01	2.99	10.37	3.80	1.05	5.78
**Frizzled-4**	3.31	5.23	4.27	3.24	0.91	2.11	9.76	13.12	20.05	1.28	9.76	7.17	1.05	8.51

**Frizzled-5**	19.84	1.21	1.00	2.97	0.91	2.53	1.81	11.33	1.50	1.28	45.73	13.07	4.21	36.48
**Frizzled-6**	34.72	1.21	2.76	1.35	0.91	1.26	1.08	11.63	1.50	1.28	41.47	51.87	12.28	48.95
**Frizzled-7**	19.43	1.21	3.52	0.81	1.52	1.26	1.08	2.09	5.51	1.28	8.23	40.90	1.40	31.62
**Galectin-3**	97.96	14.48	8.29	11.34	15.48	13.47	31.44	28.93	1.50	11.10	88.42	112.59	9.12	74.48
**GASP-1 / WFIKKNRP**	165.74	176.59	597.37	579.34	560.09	543.59	97.21	133.60	252.18	417.66	786.01	1594.75	76.50	567.29
**GASP-2 / WFIKKN**	15.71	9.65	3.27	8.91	0.91	16.42	31.08	9.54	1.50	7.26	154.58	70.00	9.83	104.28
**GCP-2 / CXCL6**	16.95	14.48	13.82	3.24	22.16	4.21	14.09	19.38	1.50	16.23	37.50	8.01	2.81	33.75
**GCSF**	29.35	37.81	59.79	20.80	45.23	16.84	30.72	54.87	65.68	46.12	27.75	30.36	13.34	17.94
**G-CSF R / CD 114**	23.56	46.66	60.04	0.81	20.64	1.26	7.95	72.17	164.45	28.61	34.45	25.72	1.05	14.90
**GDF1**	9.09	113.84	11.56	8.91	16.70	14.74	44.45	13.12	34.09	24.34	2.44	1.27	1.05	3.04
**GDF3**	61.59	29.77	26.88	29.44	39.16	47.58	115.64	49.80	74.70	37.15	30.18	22.77	17.90	18.24
**GDF5**	72.33	51.09	68.08	43.75	45.84	85.90	107.69	64.41	80.22	42.28	43.29	40.90	23.51	22.19
**GDF8**	13.23	12.07	23.61	10.26	0.91	4.63	1.08	15.51	22.06	12.81	37.50	18.55	1.05	18.85
**GDF9**	51.67	27.76	21.35	10.80	48.88	8.42	1.08	33.10	136.87	29.89	50.00	33.73	16.84	34.35
**GDF11**	48.36	24.14	43.71	26.47	3.64	9.26	15.90	100.50	57.15	22.21	83.54	60.72	16.84	45.91
**GDF-15**	138.46	101.37	114.05	135.04	193.07	50.11	174.90	294.34	269.73	394.59	14.94	41.75	63.87	152.92
**GDNF**	1.24	1.21	0.75	0.81	6.07	1.26	31.80	0.89	1.50	1.28	9.45	5.48	2.81	4.56
**GFR alpha-1**	14.47	14.08	12.81	9.45	11.84	1.26	28.91	16.70	17.55	11.96	6.10	2.53	1.05	4.56
**GFR alpha-2**	2.89	16.49	4.27	3.78	0.91	1.26	12.29	1.49	3.01	2.99	11.59	8.43	3.51	20.37
**GFR alpha-3**	9.92	49.48	31.90	18.10	7.59	1.68	18.07	21.17	33.59	14.09	15.85	14.76	4.56	13.38
**GFR alpha-4**	4.96	651.65	12.81	7.02	6.07	1.26	3.25	11.33	33.09	2.56	21.65	15.18	1.75	10.94
**GITR / TNFRF18**	4.55	22.53	22.61	11.07	1.52	2.11	21.68	34.89	347.94	22.63	48.17	38.37	1.05	31.31
**GITR Ligand / TNFSF18**	1.65	12.47	7.54	10.53	1.21	1.68	19.88	11.33	17.55	11.10	73.48	38.79	1.05	57.46
**Glucagon**	3.72	21.32	26.88	22.96	4.25	1.26	97.57	53.98	68.69	46.55	2017.47	35.42	38.60	1151.01
**Glut1**	39.27	22.93	14.57	16.75	16.39	1.26	36.14	53.08	75.71	23.91	73.17	91.08	5.26	33.44
**Glut2**	1244.12	814.97	245.43	834.57	349.41	2.53	1188.91	1485.42	888.41	1377.24	117.08	759.42	738.69	163.86
**Glut3**	16.53	11.26	9.29	10.26	13.36	1.26	32.16	10.74	19.05	11.96	8.54	8.01	2.11	4.26

**Glut5**	1.24	32.98	41.70	18.91	0.91	2.53	48.06	44.73	150.41	69.18	2.44	2.95	4.21	1.22
**Glypican 3**	1.65	12.47	9.04	9.99	0.91	1.26	34.69	37.28	17.55	11.96	31.71	75.90	6.32	26.15
**Glypican 5**	2.07	18.10	13.31	12.15	3.64	1.68	37.22	26.84	71.19	27.33	14.03	12.65	26.32	13.68
**GM-CSF**	12.40	19.71	19.59	24.85	3.64	8.42	57.10	20.28	21.06	10.68	14.94	13.92	5.61	11.25
**GM-CSF R alpha**	9.51	3.22	0.75	4.32	0.91	1.26	35.78	2.68	1.50	1.28	44.51	47.65	1.05	27.67
**Granzyme A**	74.81	104.99	53.76	42.94	2.13	1.26	66.85	149.41	156.93	61.92	214.34	94.03	24.56	186.36
**GREMLIN**	83.08	30.97	43.46	36.46	13.66	7.58	32.52	50.70	13.04	58.08	272.88	284.63	50.18	215.55
**GRO**	35.55	44.25	56.02	16.21	2.73	1.26	2.53	130.92	1.50	24.34	39.94	54.40	12.63	33.75
**GRO-a**	40.51	44.25	42.96	19.99	2.73	1.26	1.08	124.06	1.50	28.61	96.65	125.24	23.51	86.34
**Growth Hormone (GH)**	17.77	39.02	29.89	12.96	19.13	11.79	22.04	27.44	683.35	21.35	183.54	110.48	5.26	57.76
**Growth Hormone R (GHR)**	31.41	30.17	21.10	12.15	23.07	13.47	39.03	41.75	32.09	28.61	110.07	94.88	13.69	65.97
**HB-EGF**	833.68	592.92	781.75	581.77	411.65	674.12	294.16	707.97	1273.45	310.47	1920.82	1989.43	620.43	1679.99
**HCC-4 / CCL16**	20.67	25.74	26.63	9.99	20.34	13.90	21.32	31.61	9.02	22.63	50.92	40.06	1.05	34.05
**HCR / CRAM-A/B**	19.84	11.67	13.82	10.53	19.43	13.47	32.88	11.33	1.50	16.23	97.87	69.58	6.67	69.92
**Hepassocin**	160.78	69.59	51.75	30.25	57.68	30.32	49.15	104.67	115.81	49.96	57.93	85.18	4.56	37.70
**Heregulin / NDF / GGF / Neuregulin**	90.52	13.68	10.30	8.64	3.34	1.26	1.08	58.45	14.04	19.64	148.18	73.79	3.16	101.54
**HGF**	10.75	11.26	12.06	11.07	4.86	3.79	1.08	21.47	13.54	16.65	292.39	278.30	2.81	231.05
**HGFR**	32.24	24.54	31.40	17.29	4.86	2.11	28.19	114.81	84.23	31.60	55.19	22.77	2.81	35.27
**HRG-alpha**	76.05	18.10	7.54	3.78	1.82	1.26	1.08	45.93	5.51	4.27	18.60	25.72	4.56	8.82
**HRG-beta 1**	27.28	11.26	6.78	2.97	1.52	1.26	3.61	25.05	3.51	2.99	4.88	2.53	2.11	1.22
**HVEM / TNFRSF14**	17.36	38.62	28.14	5.40	4.86	1.26	8.31	43.24	23.56	6.41	10.06	10.96	1.75	8.51
**I-309**	11.16	12.07	3.01	2.70	0.91	1.26	11.93	9.84	13.04	2.56	13.72	5.48	1.05	2.13
**ICAM-1**	12.81	15.29	6.78	3.51	1.82	1.26	8.31	14.02	20.56	2.99	7.62	7.59	1.05	7.90
**ICAM-2**	61.17	21.32	7.03	4.32	2.73	1.26	6.50	36.08	44.62	1.28	4.27	15.60	4.21	10.03
**ICAM-3 (CD50)**	1.24	16.09	1.76	2.97	0.91	1.68	1.08	0.89	13.54	4.27	0.91	1.27	1.05	0.91
**ICAM-5**	1.65	12.47	8.54	4.59	3.64	2.11	3.25	4.18	41.61	2.14	8.23	20.66	11.23	9.73

**IFN-alpha / beta R1**	1.24	22.12	10.30	3.78	3.34	1.26	18.43	12.82	17.55	2.14	27.14	21.08	1.05	15.50
**IFN-alpha / beta R2**	27.28	14.48	9.04	3.51	0.91	1.26	3.25	59.35	15.54	2.56	3.35	1.27	1.05	4.56
**IFN-beta**	18.60	46.66	15.32	4.59	4.25	1.26	2.53	21.17	21.06	4.27	7.93	5.06	1.75	4.86
**IFN-gamma**	28.52	76.03	56.27	9.99	6.38	3.37	29.63	61.43	42.62	3.84	35.98	30.36	7.72	19.46
**IFN-gamma R1**	1.65	34.19	17.58	6.21	1.21	1.26	22.77	42.94	5.01	5.55	23.78	46.81	1.05	21.28
**IGFBP-1**	1.24	58.33	13.31	5.13	3.34	1.26	6.87	32.21	27.57	8.54	18.29	53.13	1.05	14.90
**IGFBP-2**	1.24	32.98	14.32	3.78	6.07	1.68	18.79	17.89	9.02	2.14	30.79	108.37	1.05	35.57
**IGFBP-3**	12.40	12.47	9.80	8.64	18.52	2.53	18.79	9.84	19.55	29.89	0.91	5.06	1.05	1.22
**IGFBP-4**	11.57	12.47	8.29	1.35	11.23	1.26	2.89	12.53	17.55	11.53	156.41	101.20	1.05	71.14
**IGFBP-6**	112.84	63.56	126.86	0.81	57.38	1.26	1.08	70.98	162.44	93.95	60.67	86.86	12.98	64.45
**IGFBP-rp1 / IGFBP-7**	25.63	60.74	50.99	8.10	9.11	1.68	2.17	77.24	8.02	1.28	75.00	83.91	1.05	54.72
**IGF-I**	11.57	13.68	9.80	2.43	3.04	1.26	2.17	15.21	1.50	1.28	73.48	55.24	1.05	41.04
**IGF-I SR**	20.67	66.77	29.89	0.81	1.52	1.26	1.08	27.44	1.50	1.28	61.28	62.83	1.05	43.17
**IGF-II**	10.33	11.67	9.04	2.16	2.73	1.26	35.41	17.30	29.08	13.24	8.54	5.48	1.05	9.73
**IGF-II R**	13.64	11.67	7.54	0.81	2.43	1.26	29.63	14.91	14.54	16.65	16.16	7.17	1.05	7.90
**IL-1 alpha**	92.59	1767.51	41.95	0.81	2.43	1.26	43.36	116.60	1215.80	49.54	149.09	89.82	7.02	118.87
**IL-1 beta**	14.05	20.92	9.80	3.24	13.36	1.26	344.39	20.28	15.04	14.95	89.94	94.45	1.05	63.24
**IL-1 F5 / FIL1delta**	45.05	25.74	82.14	5.67	36.43	2.11	62.88	34.89	69.19	39.72	98.18	88.55	4.91	66.28
**IL-1 F6 / FIL1 epsilon**	42.16	18.91	44.97	0.81	28.54	1.26	58.18	22.96	65.68	29.89	89.64	88.55	1.40	67.49
**IL-1 F7 / FIL1 zeta**	43.81	24.94	24.37	8.37	11.54	2.95	65.05	54.87	235.14	43.56	70.73	60.72	1.05	35.57
**IL-1 F8 / FIL1 eta**	13.64	21.32	17.33	0.81	2.73	1.26	38.67	22.07	23.56	13.67	76.83	43.43	1.05	53.51
**IL-1 F9 / IL-1 H1**	14.47	13.27	8.79	0.81	3.04	1.26	53.84	19.68	22.06	12.38	83.24	90.66	1.05	52.29
**IL-1 F10 / IL-1HY2**	112.84	11.67	7.79	4.32	1.52	1.68	1.08	38.77	17.05	11.53	10.98	39.22	2.11	16.11
**IL-1 R3 / IL-1 R AcP**	117.38	30.17	11.05	13.50	12.14	1.26	16.62	84.99	41.11	43.99	22.26	78.01	6.32	28.27
**IL-1 R4 /ST2**	28.52	22.53	32.91	13.77	12.45	1.26	42.28	75.75	53.65	39.72	36.59	70.42	10.18	24.02
**IL-1 R6 / IL-1 Rrp2**	3.72	14.08	12.31	6.48	1.52	1.68	13.73	21.77	9.02	70.04	45.73	52.71	1.05	27.36
**IL-1 R8**	8.27	11.67	7.28	3.51	2.43	1.26	1.08	15.21	10.03	7.26	26.83	18.98	2.11	16.11

**IL-1 R9**	2.07	28.16	4.27	4.59	7.89	2.11	6.14	11.03	11.53	14.09	73.48	55.24	1.05	44.69
**IL-1 ra**	1383.41	2576.04	261.51	313.84	214.32	53.05	188.64	357.56	332.90	1088.12	1125.66	1033.51	172.65	770.68
**IL-1 sRI**	14.47	47.06	11.81	1.35	9.11	5.47	49.15	28.93	886.40	15.37	27.75	44.70	1.05	24.02
**IL-1 sRII**	13.23	27.76	29.14	39.16	15.48	4.63	66.85	82.90	56.15	13.24	53.05	35.00	17.55	25.84
**IL-2**	13.23	73.61	40.19	14.31	8.50	3.37	51.68	86.48	136.37	21.78	22.56	10.12	3.51	12.16
**IL-2 R alpha**	1.65	18.91	9.55	10.53	5.77	1.68	50.95	15.21	16.04	4.70	92.69	15.60	7.72	41.95
**IL-2 R beta /CD122**	1.24	12.47	11.05	9.99	7.59	1.68	41.20	14.61	18.05	2.99	291.17	177.52	1.05	159.30
**IL-2 R gamma**	12.81	29.36	12.31	10.80	14.57	1.26	36.86	21.47	19.55	14.52	145.13	113.85	1.05	92.72
**IL-3**	64.07	35.80	176.35	165.56	125.38	4.21	118.53	205.77	243.66	102.07	132.93	91.50	6.32	100.33
**IL-3 R alpha**	13.23	18.50	8.54	9.99	14.57	1.26	58.54	22.07	28.08	14.09	70.13	35.00	1.05	40.74
**IL-4**	18.19	14.08	15.83	11.88	13.36	2.11	35.78	18.19	16.04	11.10	131.71	197.34	1.05	108.23
**IL-4 R**	2444.00	2288.83	2984.58	2248.75	1808.08	1.26	3144.66	2874.22	3478.93	2891.98	275.01	1905.94	1527.92	756.09
**IL-5**	35.96	35.40	44.21	21.07	31.27	1.26	49.87	78.73	79.21	27.76	35.06	48.49	1.05	28.27
**IL-5 R alpha**	2.89	17.70	18.09	5.13	4.25	1.26	54.93	18.49	21.06	16.23	122.26	71.26	1.05	33.14
**IL-6**	1.24	20.92	32.91	4.32	18.82	1.26	44.81	49.50	48.13	29.47	48.78	45.12	1.05	44.69
**IL-6 R**	1.24	29.77	26.63	1.08	5.77	1.26	40.84	28.93	19.55	13.67	89.64	94.45	8.42	107.01
**IL-7**	26.45	32.58	111.79	22.42	37.95	3.37	89.98	101.39	115.31	33.31	71.04	59.88	1.75	37.70
**IL-7 R alpha**	23.97	42.64	10.80	10.26	12.75	1.26	38.67	25.05	21.06	14.09	185.98	140.42	2.11	132.55
**IL-8**	395.97	637.57	602.14	516.95	795.97	2.95	215.74	352.19	474.79	294.24	53.66	39.22	48.08	32.53
**IL-9**	8.68	15.29	11.05	13.50	15.48	1.26	44.09	11.63	16.54	14.95	44.51	21.51	1.05	28.27
**IL-10**	7.85	12.87	16.58	11.88	3.95	1.26	42.64	26.54	21.06	13.67	66.47	43.01	2.81	28.88
**IL-10 R alpha**	6.61	11.67	11.81	10.80	1.21	1.26	44.09	14.91	4.01	12.81	56.71	53.55	1.05	23.11
**IL-10 R beta**	17.36	13.27	24.87	5.94	7.29	1.26	48.79	22.96	9.02	1.28	138.12	105.84	1.05	57.76
**IL-11**	7.85	11.26	11.81	1.62	1.21	1.26	33.61	15.81	2.51	1.28	12.50	6.33	1.05	4.86
**IL-12 p40**	3.72	23.33	25.37	0.81	5.77	1.26	39.03	29.23	1.50	1.28	46.34	39.22	1.05	41.65
**IL-12 p70**	1.24	1.21	28.89	9.72	5.46	4.21	54.57	29.23	53.65	14.09	29.88	28.25	1.05	16.72
**IL-12 R beta 1**	2.48	14.88	11.81	7.29	5.46	1.68	82.39	17.30	37.60	3.42	104.88	26.57	30.88	65.36

**IL-12 R beta 2**	63.65	9.65	9.04	10.26	1.52	1.26	44.45	31.61	15.04	11.96	68.91	24.88	1.05	26.75
**IL-13**	31.83	25.34	67.32	45.64	45.54	8.42	64.32	164.32	110.30	63.63	26.22	24.46	1.05	16.42
**IL-13 R alpha 1**	23.15	7.24	12.56	13.77	10.63	3.37	53.84	17.30	23.56	17.94	160.68	110.06	24.21	76.00
**IL-13 R alpha 2**	2.07	14.08	8.29	5.67	3.95	2.53	66.49	16.10	16.54	5.55	109.15	48.49	1.05	51.07
**IL-15**	10.33	51.49	27.38	15.40	17.61	5.89	89.98	73.66	167.45	20.50	18.60	5.90	1.05	11.55
**IL-15 R alpha**	1.24	12.07	3.01	5.94	5.16	2.11	46.98	16.10	26.57	2.99	26.83	1.27	1.75	6.99
**IL-16**	2.07	13.27	13.82	11.07	1.21	1.26	44.09	19.09	21.56	8.54	27.44	9.28	1.05	18.24
**IL-17**	5.37	11.26	11.56	12.42	3.04	1.26	47.34	18.49	28.58	9.82	15.55	6.75	12.63	11.86
**IL-17B**	1.24	2.82	23.11	18.91	7.29	1.26	70.11	25.05	62.67	2.99	14.33	2.95	2.46	10.64
**IL-17B R**	76.05	64.36	122.59	129.37	134.18	2.11	72.64	101.10	173.47	78.58	103.36	77.59	63.52	43.17
**IL-17C**	139.29	168.14	283.61	312.49	268.36	1.26	196.95	175.35	261.71	149.04	1094.26	511.90	108.08	616.54
**IL-17D**	23.97	14.48	10.05	17.29	13.36	1.26	47.70	31.01	24.07	17.08	145.74	136.62	6.67	70.23
**IL-17E**	19.43	45.05	19.59	12.15	14.27	1.26	58.54	23.86	23.06	19.22	226.84	141.26	1.05	104.89
**IL-17F**	85.15	64.76	122.84	77.25	91.98	1.26	103.35	84.10	117.82	99.08	586.92	909.54	1.05	169.95
**IL-17R**	21.49	32.58	29.89	13.77	19.13	1.26	37.58	50.10	45.12	20.07	43.60	44.28	5.61	13.68
**IL-17RC**	35.96	15.69	6.78	9.45	8.20	1.26	38.31	34.89	21.06	19.22	73.78	35.84	3.86	18.24
**IL-17RD**	83.91	16.09	2.01	9.72	6.68	1.26	37.22	26.84	17.55	6.83	72.26	34.58	3.51	29.19
**IL-18 BPa**	15.29	18.10	0.75	9.99	3.04	1.26	52.40	1.79	65.18	1.28	178.06	87.29	2.46	73.88
**IL-18 R alpha /IL-1 R5**	11.99	12.87	10.80	8.91	10.63	1.26	44.09	14.61	14.54	11.10	40.55	26.57	1.05	20.37
**IL-18 R beta /AcPL**	24.39	56.72	24.12	11.07	143.59	1.26	69.38	30.42	39.11	21.35	81.71	43.85	2.81	49.25
**IL-19**	303.80	581.26	806.62	367.32	657.84	1.26	264.16	376.95	545.98	172.10	57.01	37.95	36.15	31.92
**IL-20**	18.19	31.38	23.61	36.19	11.54	1.26	13.01	16.40	1001.71	2.99	87.50	47.65	1.05	34.35
**IL-20 R alpha**	35.96	110.62	52.75	12.15	11.23	1.26	3.25	48.91	41.61	1.28	34.15	28.25	1.05	24.02
**IL-20 R beta**	133.50	26.15	21.85	22.15	2.13	1.26	1.08	91.85	52.64	1.28	28.05	16.87	1.05	16.72
**IL-21**	2.89	12.87	3.01	0.81	0.91	1.26	21.68	3.58	15.54	1.28	7.01	7.59	12.28	7.90
**IL-21 R**	3.31	27.76	3.27	2.70	4.86	1.26	58.18	5.67	22.06	3.84	193.91	16.87	1.05	78.74
**IL-22**	7.03	2.82	3.52	5.94	2.73	1.26	1.08	3.58	14.54	2.56	500.33	5.06	1.05	73.57

**IL-22 BP**	5.37	5.23	10.30	2.70	3.04	1.26	1.08	12.82	11.03	3.84	56.71	3.80	1.05	37.09
**IL-22 R**	1.24	2.82	7.28	3.51	4.55	1.26	1.08	4.77	18.05	1.28	16.16	1.69	1.05	10.64
**IL-23**	1313.14	359.21	3151.88	2669.82	2345.71	4.63	2297.97	340.27	1171.68	380.07	378.07	645.57	715.18	423.19
**IL-23 R**	15.71	22.93	12.81	9.99	11.84	1.26	58.54	14.02	18.05	12.81	18.90	1.27	1.05	9.42
**IL-24**	33.48	16.49	17.84	20.53	20.64	1.26	55.29	26.24	66.68	22.21	57.32	59.46	16.84	93.64
**IL-26**	13.64	6.03	3.27	3.78	0.91	1.26	1.08	2.09	9.02	4.27	46.34	18.98	1.05	25.23
**IL-27**	40.09	10.46	4.27	3.24	1.52	1.26	1.08	9.24	8.52	1.71	25.31	25.30	1.05	27.67
**IL-28A**	11.16	5.23	10.30	5.67	5.16	1.26	2.53	11.33	6.52	1.28	15.55	16.45	1.05	14.59
**IL-29**	27.28	2.82	30.14	33.22	22.77	1.26	9.40	12.82	20.05	1.28	137.51	73.79	277.23	60.80
**IL-31**	33.48	1.61	9.80	12.69	5.77	1.26	3.98	11.63	3.01	1.28	44.51	44.28	1.05	22.50
**IL-31 RA**	17.77	1.21	6.78	10.80	2.73	1.26	3.25	9.24	1.50	1.28	32.93	29.10	1.05	19.15
**Inhibin A**	10.75	12.47	21.35	11.61	15.18	1.26	3.61	15.81	16.04	4.27	55.19	77.59	1.05	38.00
**Inhibin B**	22.73	12.87	53.00	43.75	51.91	1.26	3.98	17.59	724.46	1.28	102.14	253.84	15.44	52.90
**Insulin**	52.49	46.66	91.19	41.32	12.45	1.26	2.53	93.64	38.10	1.28	33.84	35.42	1.05	20.98
**Insulin R**	29.35	45.45	74.61	33.22	47.05	4.63	45.17	205.17	44.12	20.07	83.54	75.06	8.42	47.73
**Insulysin / IDE**	20.67	12.47	9.29	10.26	10.32	1.26	57.82	16.40	4.01	2.99	20.12	1.27	4.91	11.86
**IP-10**	12.40	20.92	24.87	10.26	13.66	1.26	50.59	21.77	1.50	1.28	23.78	18.55	1.05	13.38
**I-TAC / CXCL11**	54.97	32.18	165.54	86.43	111.11	1.26	104.44	82.01	299.81	48.68	545.45	607.20	38.60	304.32
**Kininostatin / kininogen**	22.32	22.12	33.41	30.52	23.07	1.26	36.50	24.45	38.10	24.77	79.58	98.67	3.86	50.16
**Kremen-1**	10.75	11.26	28.89	8.64	11.23	1.26	41.92	11.33	17.05	12.81	26.22	23.19	1.05	11.86
**Kremen-2**	1.24	8.45	4.02	4.05	2.43	1.26	1.08	13.42	7.52	2.56	15.55	6.33	1.05	8.82
**Lck**	1.24	11.26	6.53	6.48	3.34	1.26	14.45	7.16	3.51	1.28	5.79	3.37	1.05	7.30
**Latent TGF-beta bp1**	2.07	90.51	4.52	3.24	1.82	1.26	1.08	8.35	1.50	1.28	6.71	5.06	1.05	6.08
**LBP**	34.31	18.50	15.32	15.40	13.05	2.11	18.79	37.87	27.57	6.83	89.64	74.21	6.32	64.15
**LECT2**	35.13	8.85	7.79	4.32	3.34	1.26	18.07	24.16	10.03	1.28	36.59	5.48	1.05	30.40
**Lefty - A**	14.05	13.68	8.29	9.72	1.82	1.26	49.87	10.74	14.04	10.68	3.05	1.27	1.05	2.43
**Leptin R**	164.92	88.50	39.19	81.03	61.32	1.26	117.45	116.60	206.06	106.34	365.87	112.59	129.84	355.39

**Leptin (OB)**	11.16	12.47	9.80	10.80	3.04	1.26	44.45	74.85	247.17	23.49	2.74	3.80	1.05	3.95
**LFA-1 alpha**	98.37	32.58	85.41	37.27	7.59	1.68	45.17	64.41	77.71	22.63	93.91	54.40	84.92	110.05
**LIF**	26.04	11.67	10.80	12.69	5.77	1.26	3.98	17.89	22.06	4.27	51.53	48.91	1.05	47.43
**LIF R alpha**	46.71	16.49	32.66	22.42	17.00	1.26	27.46	36.98	194.03	1.71	37.81	32.05	29.83	22.19
**LIGHT / TNFSF14**	16.12	17.70	19.85	9.45	12.75	1.26	1.08	26.24	21.06	11.96	42.68	49.76	1.05	29.19
**Lipocalin-1**	220.72	66.37	211.01	137.75	75.29	1.26	216.82	222.77	614.16	99.50	212.20	315.41	629.91	192.75
**LRP-1**	35.96	11.67	9.04	10.26	12.75	1.26	23.13	27.44	16.04	11.10	124.09	104.57	1.05	86.64
**LRP-6**	44.64	3.22	7.54	9.72	9.11	1.26	5.42	25.05	17.55	2.99	85.67	43.43	1.05	117.35
**L-Selectin (CD62L)**	1.24	1.21	0.75	0.81	3.64	1.26	1.08	0.89	1.50	1.28	53.97	40.06	2.11	42.26
**Luciferase**	33.89	1.21	23.36	22.42	44.63	1.26	12.65	222.17	65.68	1.28	214.03	99.94	80.71	218.89
**Lymphotactin / XCL1**	4.13	6.84	6.78	5.94	8.20	1.26	20.96	11.93	14.54	3.42	49.09	57.35	1.05	40.43
**Lymphotoxin beta / TNFSF3**	1.24	2.41	4.27	4.05	11.23	1.26	9.40	8.05	4.01	1.28	65.25	145.90	24.21	65.97
**Lymphotoxin beta R / TNFRSF3**	1.24	1.21	6.03	1.08	2.73	1.26	5.78	7.16	1.50	1.28	62.81	77.17	1.05	44.69
**MAC-1**	2.89	9.25	14.82	11.34	6.07	2.53	7.95	15.51	26.07	3.42	28.66	13.92	1.05	15.81
**MCP-1**	1.24	8.45	42.71	10.26	13.05	1.26	21.68	156.86	79.21	1.28	25.92	17.71	2.11	12.46
**MCP-2**	1.24	1.21	10.80	1.62	4.25	1.26	5.06	13.42	56.65	1.28	54.27	1.27	1.05	22.50
**MCP-3**	13.23	10.86	8.79	11.34	4.25	1.26	10.48	14.02	33.09	15.37	324.40	374.44	23.86	207.95
**MCP-4 / CCL13**	11.99	9.65	8.29	11.07	2.73	1.26	11.20	12.53	16.04	8.54	100.00	56.93	1.05	45.60
**M-CSF**	81.01	63.56	95.71	44.56	7.59	1.26	1.08	90.66	161.44	114.45	188.73	194.39	105.98	113.70
**M-CSF R**	19.84	29.77	33.66	10.53	13.36	1.26	15.90	32.51	27.57	23.06	27.75	24.04	1.05	16.11
**MDC**	31.83	26.95	59.54	54.29	50.70	1.26	24.57	172.97	271.74	70.89	742.41	366.43	76.50	358.44
**MFG-E8**	17.77	13.27	12.56	8.37	12.14	1.26	1.08	14.02	28.58	13.24	953.09	30.78	1.05	20.07
**MFRP**	10.75	12.47	9.04	3.51	2.43	1.68	73.36	14.61	14.04	2.56	40.25	51.44	1.05	39.83
**MIF**	13.64	17.70	31.90	8.91	8.50	2.95	59.27	31.31	35.10	8.54	16.16	21.51	1.05	13.07
**MIG**	31.41	30.17	51.25	13.50	17.91	5.47	72.27	212.63	119.32	4.70	32.62	28.25	10.18	11.86
**MIP-1a**	364.14	108.61	66.32	70.22	77.71	4.21	105.88	201.89	167.45	162.71	128.97	169.93	457.25	85.73

**MIP-1b**	30.17	12.87	15.07	15.12	20.34	2.11	54.21	20.58	46.13	21.35	21.95	8.86	1.05	21.89
**MIP-1d**	7.85	10.86	8.04	8.10	14.57	1.26	32.88	10.14	9.53	13.24	41.47	44.70	1.05	27.97
**MIP 2**	21.91	12.47	8.04	8.10	1.21	2.11	7.23	6.56	7.52	5.55	34.76	16.45	1.05	27.97
**MIP-3 alpha**	97.13	12.87	8.04	10.53	6.38	1.26	17.35	14.31	7.52	3.42	171.35	223.48	1.05	140.46
**MIP-3 beta**	40.51	12.47	5.28	4.86	4.55	1.26	10.48	22.07	6.52	1.28	41.47	59.88	1.05	33.44
**MMP-1**	2.48	4.83	6.78	2.70	0.91	1.26	2.17	1.79	17.05	3.84	22.56	72.53	1.05	39.52
**MMP-2**	11.57	9.25	11.30	8.91	6.07	1.68	20.96	10.44	35.60	5.55	75.92	72.53	1.05	83.30
**MMP-3**	7.03	5.23	10.30	4.59	9.11	1.26	9.40	15.51	16.54	2.56	80.49	46.81	1.05	70.53
**MMP-7**	30.17	13.27	28.39	11.88	2.73	2.11	5.42	5.67	150.41	9.40	27.14	12.23	4.91	15.50
**MMP-8**	33.07	13.68	23.86	17.02	14.57	1.26	33.61	24.75	165.95	8.97	37.20	27.41	1.05	22.50
**MMP-9**	13.23	2.41	10.30	4.59	2.13	1.26	20.60	17.89	29.08	1.28	16.46	1.27	1.05	17.02
**MMP-10**	25.63	10.06	6.28	9.18	11.54	1.26	63.24	14.61	12.03	3.42	33.84	37.95	2.46	29.49
**MMP-11 /Stromelysin-3**	13.23	4.42	4.52	3.24	5.16	1.26	59.99	9.84	3.51	1.28	23.48	13.07	3.51	17.33
**MMP-12**	14.47	1.21	3.01	0.81	5.46	1.26	56.74	4.77	1.50	1.28	23.78	34.16	1.05	16.11
**MMP-13**	9.09	22.93	11.05	10.53	7.89	1.26	22.77	34.89	46.13	3.42	47.87	75.48	1.05	36.79
**MMP-14**	2.89	29.36	19.59	4.59	10.63	1.26	2.17	25.65	319.87	1.28	28.05	27.83	1.05	18.54
**MMP-15**	1.24	13.68	4.27	1.89	6.98	1.26	2.53	15.21	186.51	1.28	108.24	125.24	1.05	77.52
**MMP-16 / MT3-MMP**	36.79	12.47	5.78	8.37	1.52	1.68	29.63	25.05	15.54	2.99	96.96	102.04	1.05	72.96
**MMP-19**	54.15	22.93	14.57	19.18	21.25	1.26	44.09	25.65	61.17	1.28	147.26	99.94	2.11	90.60
**MMP-20**	47.53	25.74	19.85	25.66	30.96	7.58	18.79	25.65	64.17	1.28	196.35	96.56	5.26	97.89
**MMP-24 / MT5-MMP**	27.28	10.06	17.33	14.58	4.25	1.26	13.01	27.14	40.61	5.12	41.77	48.49	1.05	23.11
**MMP-25 / MT6-MMP**	13.64	1.21	8.29	11.07	2.13	1.26	7.95	9.54	15.04	1.28	9.15	6.75	1.05	7.90
**Musk**	42.99	1.21	30.65	16.48	4.86	1.26	6.14	38.17	56.65	1.28	34.76	28.67	1.05	17.63
**MSP alpha Chain**	33.07	35.40	35.42	12.42	4.55	1.26	37.94	41.45	70.19	3.84	58.54	97.83	1.05	50.77
**MSP beta-chain**	25.63	13.27	15.07	6.75	16.70	1.26	1.81	16.40	70.69	1.28	22.26	13.49	14.74	10.03
**NAP-2**	11.99	23.33	22.36	0.81	2.73	1.26	1.45	25.94	28.58	1.28	38.42	35.84	1.05	28.88
**NCAM-1 / CD56**	128.96	16.89	17.58	10.26	3.95	1.68	14.09	46.52	22.06	7.69	107.32	85.18	5.26	52.29

**Neuritin**	22.73	10.06	12.81	8.37	14.27	3.37	1.08	12.23	350.95	5.98	18.29	10.12	6.67	13.07
**NeuroD1**	88.45	10.86	9.55	4.05	5.77	1.26	1.08	37.58	16.04	1.71	94.52	56.50	1.05	33.75
**Neuropilin-2**	6.20	8.85	11.30	4.32	3.04	1.68	1.08	14.02	16.04	3.84	91.77	130.30	2.46	48.64
**Neurturin**	22.73	28.56	132.64	25.66	20.64	3.37	7.23	38.47	195.53	20.07	36.59	72.11	33.34	32.53
**NGF R**	2.48	12.87	20.85	7.56	7.89	1.26	1.08	14.31	18.05	3.42	30.18	39.64	13.69	33.44
**NOV / CCN3**	20.67	19.71	13.31	0.81	0.91	1.26	1.08	19.09	5.51	3.84	56.40	43.43	1.05	53.20
**NRG1 Isoform GGF2**	191.37	13.27	14.57	6.21	2.73	1.26	1.08	76.94	15.04	5.98	68.30	55.66	1.05	43.47
**NRG1-alpha / HRG1-alpha**	92.17	15.29	12.56	5.40	3.04	1.26	1.08	63.22	26.07	5.55	38.72	25.30	2.81	27.36
**NRG1-beta1 / HRG1-beta1**	19.43	28.16	30.65	9.18	5.77	1.26	160.81	24.45	36.10	21.78	9.76	5.90	2.81	13.98
**NRG2**	601.39	69.99	353.70	291.97	412.86	16.42	284.40	262.73	1128.06	347.62	90.25	22.77	7.72	25.84
**NRG3**	108.71	64.36	42.20	33.22	33.70	2.53	130.82	41.75	86.23	67.47	30.49	17.29	10.53	21.59
**NT-3**	20.25	24.54	21.85	4.32	15.18	1.26	1.08	15.51	18.55	3.84	37.50	32.05	1.05	17.94
**NT-4**	91.76	30.97	18.84	23.77	31.27	1.26	18.43	84.99	145.90	19.22	37.50	2.11	4.91	16.42
**Orexin A**	15.71	6.44	30.90	7.56	14.57	1.26	1.08	14.02	18.05	2.56	32.62	29.52	1.05	23.41
**Orexin B**	13.23	1.21	10.30	11.61	10.32	1.26	54.93	9.84	9.53	9.82	0.91	1.27	1.05	0.91
**OSM**	147.14	1.21	406.45	135.31	98.05	1.26	166.59	174.16	346.44	337.80	243.61	422.93	611.66	122.82
**Osteoactivin / GPNMB**	22.32	1.21	40.95	16.48	15.48	1.26	61.07	78.73	127.85	79.43	44.21	70.42	116.51	25.54
**Osteocrin**	133.09	13.27	9.29	11.61	12.75	1.26	85.28	34.59	20.56	11.96	52.44	38.37	1.05	49.55
**Osteoprotegerin / TNFRSF11B**	633.63	802.10	565.97	1049.83	923.77	1.26	520.01	2211.28	947.07	842.57	1120.78	2132.80	2747.03	966.47
**OX40 Ligand / TNFSF4**	13.64	14.88	31.40	17.56	27.93	1.26	64.32	60.84	61.17	20.07	155.49	180.90	8.42	100.63
**PARC / CCL18**	4.13	10.86	12.81	8.91	7.89	1.26	10.48	14.31	16.54	2.14	98.48	59.46	1.05	65.06
**PD-ECGF**	1.24	5.23	11.05	4.05	2.73	6.74	10.12	14.31	5.51	1.28	26.53	23.19	1.05	86.04
**PDGF R alpha**	1.65	1.21	15.57	3.51	1.21	1.26	10.12	5.07	9.53	1.28	5.79	3.80	1.05	4.56
**PDGF R beta**	16.12	46.26	35.17	11.88	7.59	1.26	39.75	39.07	40.11	6.41	60.06	53.97	1.05	37.09
**PDGF-AA**	5.37	17.30	10.80	31.87	3.34	1.26	16.62	32.51	20.05	1.28	67.99	24.04	1.05	59.89
**PDGF-AB**	1.24	21.72	24.37	1.89	6.07	1.26	1.08	46.22	134.87	1.28	48.48	23.19	1.05	29.19

**PDGF-BB**	23.56	10.86	12.31	9.99	5.46	1.26	53.12	22.66	23.06	3.84	67.38	24.04	1.05	36.79
**PDGF-C**	35.96	3.22	8.54	4.86	3.64	1.26	6.87	18.79	5.51	1.28	96.04	24.04	1.05	35.87
**PDGF-D**	16.12	1.21	7.28	1.08	2.43	1.26	1.08	3.58	1.50	1.28	8.84	2.53	1.05	5.47
**PECAM-1 /CD31**	21.91	9.25	53.26	95.61	117.48	2.11	1.08	16.70	10.53	5.12	26.83	41.75	1.75	15.20
**Pentraxin3 / TSG-14**	160.78	7.24	9.04	9.72	15.18	1.26	1.08	50.10	9.53	2.99	70.43	34.16	1.05	37.39
**Persephin**	26.87	8.45	14.57	10.26	13.36	1.26	2.53	17.89	4.51	3.42	16.16	3.80	1.05	7.90
**PF4 / CXCL4**	10.75	6.03	7.28	25.93	0.91	3.79	1.81	30.42	95.76	14.09	47.87	14.76	4.56	47.73
**PlGF**	3.72	13.68	16.58	12.15	4.25	3.37	1.08	15.21	16.54	5.12	22.56	35.84	1.05	16.72
**PLUNC**	2.48	4.83	17.08	9.45	4.86	1.26	1.08	11.33	11.03	2.99	15.85	16.02	1.75	9.42
**Pref-1**	7.44	10.86	14.32	8.10	0.91	1.26	1.08	14.31	13.04	11.96	60.67	89.39	1.05	38.61
**Progranulin**	7.85	6.03	11.56	6.48	1.82	1.26	1.08	22.07	6.02	5.12	5.18	2.11	1.05	3.04
**Prolactin**	3.31	11.67	25.87	5.40	2.43	1.26	1.08	22.37	13.54	5.12	45.43	58.19	1.05	31.31
**P-selectin**	122.76	28.96	34.42	27.82	0.91	1.68	16.26	121.97	62.67	38.86	78.36	53.13	1.75	48.64
**RAGE**	69.44	41.03	32.41	15.40	5.77	2.11	1.08	59.35	244.66	17.08	101.53	146.74	1.75	72.36
**RANK / TNFRSF11A**	18.19	15.69	13.06	14.85	7.59	2.11	7.23	34.59	110.30	9.82	61.59	55.66	1.05	35.87
**RANTES**	30.17	34.19	18.34	15.94	11.23	1.26	1.08	22.96	36.10	15.80	97.57	51.02	1.05	57.46
**RELM beta**	13.23	11.26	10.80	10.26	12.14	1.26	1.08	13.12	16.54	4.70	57.01	18.55	1.05	47.73
**RELT / TNFRSF19L**	52.08	26.55	24.87	27.01	32.18	1.26	6.14	33.40	77.71	2.99	27.44	26.57	1.05	23.71
**ROBO4**	1.24	3.22	17.84	18.37	11.54	1.26	139.13	9.54	49.13	6.83	157.93	308.24	1374.22	132.25
**S100 A8/A9**	4.13	14.08	24.12	14.85	48.27	4.63	138.04	41.15	55.15	1.28	65.86	42.17	103.17	51.07
**S100A10**	1.24	1.21	14.07	19.72	31.88	1.26	169.84	3.28	21.06	1.28	8.23	5.90	3.16	5.17
**SAA**	8.27	11.26	20.85	23.23	336.36	1.68	1.08	71.27	188.51	5.98	47.26	28.25	38.25	19.46
**SCF**	1.24	1.21	13.82	13.77	15.48	1.26	28.19	40.26	44.62	1.28	50.31	56.50	1.05	34.96
**SCF R /CD117**	1.24	1.21	2.26	13.50	13.36	1.26	30.72	22.37	21.06	1.28	126.53	110.48	1.05	80.26
**SDF-1 / CXCL12**	1.24	6.84	10.05	9.99	10.93	1.26	22.04	12.82	18.05	14.09	75.92	32.05	1.05	75.40
**sFRP-1**	64.89	18.50	11.81	11.07	16.70	1.26	7.59	21.77	35.10	13.24	17.68	13.07	3.16	7.60
**sFRP-3**	293.46	82.86	114.80	94.53	424.09	1.26	1.08	161.34	1073.91	73.45	73.78	91.92	1.05	63.24

**sFRP-4**	23.56	12.07	12.31	14.31	16.39	1.26	66.85	13.72	20.56	14.95	19.21	9.28	1.05	9.73
**sgp130**	39.27	29.77	30.90	76.16	82.27	1.26	79.86	65.31	80.22	55.09	54.88	45.12	1.05	45.30
**SIGIRR**	4701.60	5483.14	1991.81	3195.14	3858.11	1.26	6634.43	5739.19	7120.30	9644.50	1714.10	1448.43	739.40	1684.55
**Siglec-5/CD170**	1.24	1.21	10.30	11.34	12.45	1.26	66.49	13.42	15.04	14.95	17.38	7.59	1.05	14.29
**Siglec-9**	1.24	1.21	166.30	412.96	402.23	1.26	214.66	301.80	265.22	241.28	789.97	579.37	86.33	571.55
**SLPI**	1.24	1.21	8.29	9.45	13.96	1.26	47.34	15.21	17.55	14.95	7.62	1.69	1.05	6.99
**Smad 1**	52.08	1.21	0.75	8.91	1.52	1.26	1.08	29.52	2.01	1.71	1.83	1.27	1.05	0.91
**Smad 4**	14.05	1.21	0.75	31.06	6.38	1.26	13.37	13.72	5.51	3.84	0.91	1.27	1.05	0.91
**Smad 5**	33.89	1.21	2.76	16.21	9.41	1.26	29.27	17.30	5.51	4.27	4.88	3.37	1.05	31.31
**Smad 7**	38.44	16.09	18.84	19.45	23.68	1.26	10.84	18.19	18.05	5.12	45.73	84.33	5.26	69.32
**Smad 8**	291.40	79.65	60.04	51.86	126.59	8.00	1.08	24.75	19.55	7.26	50.31	56.93	9.47	102.15
**SMDF / NRG1Isoform**	18.19	13.68	14.07	14.04	21.55	1.26	1.08	19.98	27.07	3.84	2.44	7.17	1.05	5.17
**Soggy-1**	16.95	20.92	4.77	4.32	2.73	1.68	1.08	22.96	18.55	2.99	24.70	35.42	1.05	56.85
**Sonic Hedgehog (Shh N-terminal)**	18.19	24.94	6.78	6.48	2.73	1.68	1.08	21.17	29.08	1.71	9.76	40.06	1.05	21.59
**SPARC**	37.61	13.27	5.53	4.86	2.73	1.26	1.08	20.58	13.04	3.84	10.06	1.27	1.05	15.81
**Spinesin**	30.17	4.42	10.55	1.08	0.91	1.26	1.08	18.79	20.05	5.55	3.96	1.27	1.05	8.21
**TACI / TNFRSF13B**	23.97	9.25	28.64	7.02	3.04	4.63	1.08	21.77	73.20	11.10	87.81	159.81	12.98	84.52
**Tarc**	12.81	5.63	9.55	4.59	6.38	1.68	10.84	115.41	692.38	35.02	163.42	116.38	1.05	65.36
**TCCR / WSX-1**	46.71	6.44	21.10	19.72	0.91	1.26	25.66	0.89	2.51	7.26	75.00	118.07	1.05	121.61
**TECK / CCL25**	12.81	12.47	11.30	11.34	0.91	1.26	1.08	1.79	5.01	2.99	40.25	3.80	1.05	44.39
**TFPI**	16.12	3.62	9.80	10.53	0.91	1.26	1.08	5.37	4.51	4.27	6.40	1.27	1.05	3.95
**TGF-alpha**	47.53	15.29	12.06	12.96	5.77	1.26	1.08	26.54	19.55	1.28	85.37	86.44	4.91	50.16
**TGF-beta 1**	24.80	28.16	35.42	26.20	4.25	1.26	1.08	59.94	47.13	1.28	59.76	89.39	23.16	51.68
**TGF-beta 2**	20.25	14.48	32.91	22.96	15.79	1.26	1.08	15.51	41.11	1.28	54.88	63.25	1.05	67.49
**TGF-beta 3**	22.32	47.06	33.41	11.34	2.73	1.26	1.08	36.08	41.61	14.95	60.67	72.11	1.05	55.94
**TGF-beta 5**	125.24	23.33	17.58	16.21	15.79	1.26	1.08	96.32	29.58	16.23	79.58	136.20	36.85	73.57

**TGF-beta RI / ALK-5**	198.81	37.41	28.89	40.51	47.96	1.26	1.08	91.85	56.65	38.43	17.07	10.54	1.05	151.70
**TGF-beta RII**	27.69	47.47	30.65	15.12	2.73	1.26	1.08	35.49	32.09	5.12	78.05	96.14	6.67	77.83
**TGF-beta RIIb**	71.09	16.09	12.56	16.21	11.84	1.26	1.08	54.57	22.06	5.55	187.51	142.95	1.05	188.19
**TGF-beta RIII**	8.68	11.67	9.29	11.61	5.46	1.26	1.08	11.63	14.04	2.99	158.85	219.27	12.28	116.74
**Thrombopoietin (TPO)**	19.43	11.67	12.31	11.07	40.98	1.26	69.74	16.70	49.13	16.65	78.36	99.09	12.63	56.85
**Thrombospondin (TSP)**	776.23	665.33	879.47	330.05	179.41	58.95	277.53	410.94	1627.91	936.95	344.53	317.94	289.86	152.31
**Thrombospondin-1**	21.49	13.68	79.38	29.71	17.00	4.21	66.13	25.05	51.14	32.03	5.49	7.59	1.05	3.65
**Thrombospondin-2**	9746.67	9142.46	9825.17	11120.34	12527.55	7.16	12136.33	11511.19	18113.09	15229.89	1124.44	2857.64	1264.38	1401.82
**Thrombospondin-4**	12.40	23.33	9.55	10.80	16.09	1.26	78.78	12.53	2849.22	16.23	10.06	5.48	1.05	11.25
**Thymopoietin**	13.23	18.50	10.55	9.45	11.23	1.26	76.97	13.42	3.51	12.81	152.75	199.87	1.05	71.44
**Tie-1**	2.89	8.85	22.61	2.43	0.91	1.26	1.08	21.17	22.06	16.65	5.79	11.81	1.05	13.07
**Tie-2**	4.96	9.65	28.39	5.40	1.82	1.68	1.08	23.56	20.05	15.80	16.16	44.70	1.05	41.65
**TIMP-1**	16.53	6.84	16.08	12.69	17.61	1.26	10.48	27.44	64.68	31.17	18.90	47.23	1.05	42.56
**TIMP-2**	1.24	16.09	13.57	9.72	0.91	1.26	108.41	13.12	1.50	3.84	16.16	25.30	1.05	8.51
**TIMP-3**	3.31	27.35	15.83	10.53	2.43	1.68	130.82	17.00	9.53	5.12	2.13	5.90	1.05	11.25
**TIMP-4**	15.29	38.62	14.07	9.99	6.68	1.68	121.42	15.51	15.04	7.69	14.03	18.13	1.05	22.50
**TL1A / TNFSF15**	15.29	1.21	1.26	2.16	0.91	1.26	1.08	18.79	1.50	2.56	14.03	10.54	6.67	16.72
**TLR1**	40.92	8.05	5.02	5.40	2.73	1.26	1.08	17.00	11.03	6.83	13.42	10.54	1.05	18.85
**TLR2**	29.76	8.45	5.53	4.05	5.77	1.26	10.12	17.89	15.54	8.11	10.06	4.64	4.91	9.73
**TLR3**	37.61	16.49	10.05	10.80	0.91	1.26	145.27	16.40	25.07	16.23	17.07	11.39	15.79	15.81
**TLR4**	13.23	12.87	11.05	11.07	4.55	2.11	168.76	11.63	22.06	15.37	3.96	1.27	1.05	7.30
**TMEFF1 / Tomoregulin-1**	157.89	33.79	30.65	132.61	166.05	2.11	431.48	602.10	549.99	228.47	309.16	207.46	1871.13	625.06
**TMEFF2**	92.59	2.82	18.09	22.69	0.91	1.26	1.08	20.58	19.05	4.70	159.46	188.06	1.05	166.90
**TNF-alpha**	1.24	1.21	3.27	4.05	0.91	1.26	1.08	0.89	1.50	1.28	97.26	68.73	13.34	70.23
**TNF-beta**	48.77	1.21	35.67	15.94	12.75	1.26	7.59	150.00	84.23	1.28	52.14	41.32	9.47	34.05
**TNF RI / TNFRSF1A**	14.88	12.47	15.07	13.50	20.64	1.26	149.97	18.79	27.57	15.37	21.65	10.96	2.81	4.56
**TNF RII / TNFRSF1B**	3311.17	2744.99	2371.89	1369.89	1465.65	1.26	1318.29	2165.36	4750.37	4446.45	289.65	589.07	323.90	266.32

**TRADD**	20.25	19.31	22.36	19.45	20.64	1.26	130.09	10.44	179.49	73.03	11.28	7.17	1.05	2.43
**TRAIL / TNFSF10**	2.48	2.01	9.55	0.81	0.91	1.26	1.08	6.56	4.51	2.99	21.34	20.66	1.05	19.15
**TRAIL R1 / DR4 / TNFRSF10A**	8.27	2.41	13.82	4.32	5.16	1.26	1.08	10.44	13.04	2.14	46.95	45.12	13.69	43.78
**TRAIL R2 / DR5 / TNFRSF10B**	15.71	1.21	14.57	7.83	17.61	1.26	8.67	14.91	5.51	1.28	41.16	38.79	5.97	54.11
**TRAIL R3 / TNFRSF10C**	4.96	2.41	10.55	0.81	0.91	1.26	1.08	3.28	19.05	4.27	37.50	42.59	1.05	25.84
**TRAIL R4 / TNFRSF10D**	6.61	2.41	12.56	2.43	1.52	1.26	2.89	7.46	21.56	2.56	48.48	45.54	2.81	40.13
**TRANCE**	18.60	1.21	17.08	5.67	16.09	1.26	9.76	12.82	142.89	1.28	19.51	10.96	10.53	9.73
**TREM-1**	14.47	7.64	21.35	6.48	6.98	2.11	1.08	34.29	18.05	14.95	6.71	1.27	20.70	6.99
**TROY / TNFRSF19**	28.52	3.62	13.06	5.94	6.98	1.26	1.08	24.16	7.02	5.98	3.35	1.27	3.86	2.43
**TSG-6**	1.65	1.21	11.05	3.24	3.95	1.26	1.08	159.84	463.26	12.38	6.71	1.27	5.26	4.56
**TSLP**	1.24	1.21	10.05	3.51	0.91	1.26	1.08	17.59	16.54	1.28	0.91	1.27	1.05	0.91
**TWEAK / TNFSF12**	16.95	6.03	10.55	4.86	7.89	2.95	1.08	28.93	17.05	3.42	7.62	1.27	11.93	6.08
**TWEAK R / TNFRSF12**	59.52	2.41	9.29	9.18	11.23	1.26	110.22	26.54	11.53	5.98	0.91	1.27	1.05	0.91
**Ubiquitin+1**	27.69	7.64	16.58	12.69	19.43	2.11	63.96	17.30	38.60	17.94	31.40	15.18	23.51	22.80
**uPA**	4.13	1.21	324.31	462.66	251.36	1.68	703.59	106.46	379.53	274.59	178.67	318.78	707.46	383.97
**uPAR**	1.24	1.21	18.34	4.05	0.91	1.26	1.08	7.16	21.56	5.98	16.77	1.27	1.05	13.07
**Vasorin**	1.24	1.21	19.85	7.29	4.86	1.26	1.08	6.86	146.90	8.11	21.34	1.27	34.39	24.63
**VCAM-1 (CD106)**	1.24	1.21	13.31	5.40	2.13	1.26	1.08	12.82	22.56	6.41	10.37	1.27	1.05	9.73
**VE-Cadherin**	7.03	16.49	12.56	1.08	0.91	1.26	1.08	4.47	15.04	3.84	44.51	21.51	14.04	25.84
**VEGF**	13.23	9.25	10.05	3.24	1.82	1.26	1.08	4.18	9.02	1.28	17.07	20.24	3.51	11.55
**VEGF R2 (KDR)**	11.57	51.49	14.32	2.43	3.34	1.26	9.40	12.82	8.52	1.28	29.88	15.60	5.26	20.07
**VEGF R3**	13.64	2.82	10.80	0.81	0.91	1.26	1.08	5.96	7.52	4.70	14.94	7.59	1.05	3.04
**VEGF-B**	30.17	1.21	9.80	0.81	0.91	1.26	1.08	6.56	1.50	1.28	7.32	5.06	1.05	0.91
**VEGF-C**	16.95	1.21	10.05	3.51	5.16	1.26	1.08	8.05	2.01	1.28	9.15	7.17	1.05	1.52
**VEGF-D**	5.37	1.61	10.55	0.81	0.91	1.26	125.40	19.68	9.02	4.27	19.82	10.54	13.34	2.43
**VEGI / TNFSF15**	9.09	2.01	11.56	2.43	1.82	1.26	115.64	19.98	9.53	1.28	14.03	10.54	1.05	3.95

**WIF-1**	9.51	1.21	11.05	3.78	8.50	1.26	133.71	29.82	5.01	1.28	46.95	43.43	8.42	50.77
**WISP-1 / CCN4**	10.75	1.21	9.80	9.18	13.36	1.26	100.10	14.02	18.05	13.67	0.91	1.27	1.75	0.91
**XEDAR**	317.44	177.80	206.74	257.66	242.55	1.26	1057.74	93.64	1233.34	170.39	1.52	1.27	1.05	0.91
